# Global and regional knowledge of antibiotic use and resistance among healthcare students: A systematic review and meta‐analysis

**DOI:** 10.1002/bcp.70575

**Published:** 2026-04-23

**Authors:** Asa Auta, Erick Wesley Hedima, Emmanuel O. Adewuyi, Shalkur David, Emmanuel Agada David, Lomikga Balachandran, Enoche Florence Oga, Davies Adeloye, Barry Strickland‐Hodge

**Affiliations:** ^1^ Faculty of Health, Social Care and Medicine Edge Hill University Ormskirk UK; ^2^ Department of Clinical Pharmacy and Pharmacy Practice, Faculty of Pharmaceutical Sciences Gombe State University Gombe Nigeria; ^3^ Curtin School of Population Health, Faculty of Health Sciences Curtin University Perth Australia; ^4^ Curtin Medical Research Institute Curtin University Perth Australia; ^5^ Curtin enAble Institute Curtin University Perth Australia; ^6^ Department of Clinical Pharmacy and Pharmacy Practice University of Jos Jos Nigeria; ^7^ School of Pharmacy and Biomedical Sciences University of Lancashire Preston UK; ^8^ School of Health & Life Sciences Teesside University Middlesbrough UK; ^9^ School of Healthcare University of Leeds Leeds UK

**Keywords:** antibiotic resistance, antibiotics, bacterial infection, healthcare students

## Abstract

**Aims:**

We synthesized and analysed existing evidence on healthcare students' knowledge of antibiotic use and resistance to provide pooled global and regional estimates.

**Methods:**

The PubMed®, Embase® and CINAHL databases were searched for studies published between January 2015 and October 2025 that reported the knowledge of antibiotic use and resistance among healthcare students. Pooled estimates and 95% confidence interval (CI) of correct knowledge were determined using random‐effects meta‐analysis.

**Results:**

Of the 8623 articles identified, 131 studies with data from 43 countries met the inclusion criteria. Most healthcare students correctly understood that antibiotics are effective against bacterial infections (88.7%, 95% CI: 87.0–90.5) but ineffective against viruses (70.0%, 95% CI: 65.6–74.4). However, only 57.9% (95% CI: 51.5–64.3) knew that antibiotics are ineffective against colds and flu, while just over half (51.5%, 95% CI: 41.6–61.4) of the students correctly knew that antibiotics do not speed up recovery from common colds and flu. Significant regional differences were observed in the understanding that antibiotics do not speed up recovery from common colds and flu, ranging from 41.3% (95% CI: 33.5–49.1) in South Asia to 98.0% (85% CI: 94.5–99.3) in North America.

**Conclusions:**

Although our findings indicate a substantial level of antibiotic knowledge across many of the evaluated outcomes, there are significant knowledge gaps in understanding of the ineffectiveness of antibiotics against the common cold and flu. These knowledge gaps have important implications for the rational use of antibiotics and the prevention of resistance.

## 1 INTRODUCTION

The introduction of antibiotics in clinical practice has revolutionized modern medicine, enabling the treatment of previously fatal infections and greatly enhancing public health outcomes. Today, antibiotics are among the most commonly prescribed lifesaving medications.^1^, ^2^ However, the rapid emergence of antibiotic resistance now threatens these advancements, posing major global health and developmental challenges.^3^, ^4^ In 2021, resistant bacterial infections were estimated to be responsible for approximately 1.14 million deaths worldwide.^5^ Antibiotic‐resistant bacteria account for over 2.8 million infections and more than 35 000 deaths annually in the USA.^6^ Europe faces a similar situation, with a comparable number of deaths attributed to antibiotic‐resistant infections.^7^ While reliable estimates for developing countries are scarce, it is believed that the burden of antibiotic‐resistant bacterial infections results in even higher mortality rates in these regions.

A major driver of antibiotic resistance is the overprescription or unnecessary supply of antibiotics by healthcare professionals, particularly for conditions that do not require them, such as viral infections like the common cold or flu. A global systematic review revealed that approximately two‐thirds of patients presenting with symptoms of upper respiratory tract infections in community pharmacies were supplied with antibiotics.^1^ For conditions where antibiotic use is justified, the inappropriate application of broad‐spectrum antibiotics, when a narrow‐spectrum alternative would suffice, has been reported.^8^, ^9^ This practice has further exacerbated the rising threat of antibiotic resistance.

The overuse of antibiotics by healthcare professionals is often linked to a lack of knowledge regarding the rational use of these medications and their potential risks.^10^, ^11^, ^12^ To address this issue and combat antibiotic resistance, it is essential to implement a strategy that includes the education and training of future healthcare providers, who will be responsible for prescribing, supplying and administering antibiotics. This strategy should encompass the development of comprehensive curricula aimed at equipping healthcare students about judicious antibiotic prescribing and supply. Over the past decade, numerous studies, which are included in this systematic review, have examined healthcare students' knowledge regarding antibiotic use and resistance, addressing various outcomes. However, no systematic review has yet been conducted to provide pooled global and regional estimates of these outcomes. Conducting such a review is crucial for identifying knowledge gaps and pinpointing areas where targeted curricular and training interventions are necessary. This systematic review aims to synthesize and analyse existing evidence on healthcare students' understanding of antibiotic use and resistance. It specifically focuses on healthcare students' general knowledge of antibiotics and common misconceptions, rather than broader aspects of antimicrobial stewardship or students' future prescribing competence.

## 2 METHODS

### 2.1 Information sources and search strategy

This review was performed in accordance with the Preferred Reporting Items for Systematic Reviews and Meta‐Analyses (PRISMA) 2020 guidelines.^13^ The study protocol was registered in the PROSPERO international prospective register of systematic reviews (CRD 420251076347).

We searched the PubMed®, Embase® (via Ovid) and CINAHL (via EBSCO) databases for original research articles published in the English language from January 2015 to October 2025. This timeframe was selected to capture the most recent and relevant evidence on healthcare students' knowledge of antibiotics, reflecting current educational contexts and curriculum developments. It also corresponds with the increased global focus on antimicrobial resistance following the publication of the World Health Organization's Global Action Plan on Antimicrobial Resistance in 2015,^14^ which prompted greater institutional efforts in education, policy and research. Our searches were aimed at retrieving articles that reported the assessment of actual knowledge of antibiotic use and resistance among healthcare students. The search strategy employed Medical Subject Headings (MeSH), truncations and keywords along with their synonyms, including antibiotics, healthcare, students and knowledge which were combined using Boolean operators (Table S1). Additional articles were identified by checking reference lists of eligible studies and by Google Scholar citation tracking.

### 2.2 Eligibility criteria and selection process

Search results were imported into a citation manager (EndNote version 21.4), and duplicates were removed. Two reviewers, AA and EWH, independently screened studies against the inclusion and exclusion criteria. Any discrepancies were resolved through consensus. In this systematic review, we included observational and interventional studies involving healthcare students that assessed and reported the percentage of correct/incorrect responses to knowledge questions on antibiotic use and antibiotic resistance, based on at least one of the eleven outcomes listed in Table S2, which were informed mainly by the European Union and WHO multi‐country knowledge assessment areas.^15^, ^16^ Additionally, we gathered data on the percentage of healthcare students who are aware of antibiotic resistance and those who recognize it as a global public health threat.

Research papers were included in this review if they focused on students enrolled in programmes designed to prepare them for diverse roles in healthcare, such as medicine, nursing, pharmacy, veterinary medicine and dentistry. We excluded studies that assessed antibiotic knowledge among qualified healthcare professionals. Furthermore, quantitative studies solely focussed on healthcare students' perceived knowledge, attitude and practice concerning antibiotic use and resistance were excluded from the review. Studies that employed qualitative data collection methods, as well as reviews, editorials, opinion articles and conference abstracts, were excluded.

### 2.3 Data extraction

Data from each eligible study were extracted using a piloted template designed in Microsoft® Excel® for Microsoft 365 MSO (Microsoft Office Suite) Version 2308. The data were extracted by AA and checked by EOA, SD and EFO as independent second reviewers. Any discrepancy in data extraction was resolved by consensus. The data extracted included author, year of publication, study country, study region, sample size, percentages of correct responses for assessed knowledge outcomes and types of validity and reliability measures. The regional location of each study was categorized according to the World Bank classification criteria, which include Europe and Central Asia, the Middle East and North Africa, sub‐Saharan Africa, South Asia, Latin America and the Caribbean, East Asia and Pacific and North America.

### 2.4 Study risk of bias assessment

The risk of bias of each study was assessed by AA using the Medical Education Research Quality Instrument (MERSQI) and subsequently verified by EAD, LB and SD as independent second reviewers. The MERSQI tool is designed to evaluate the methodological quality of observational, quasi‐experimental and experimental studies in medical education, making it ideal for assessing the quality of quantitative studies that examine participants' knowledge.^17^ This tool included 10 items across 6 domains: study design, sampling, type of data (subjective or objective), validity, data analysis and outcomes. Each domain has a maximum score of 3, producing a maximum possible MERSQI score of 18 and a potential range of 5–18. The risk of bias of each study was classified as low (total score: 13 to 18), moderate (total score: 10 to 12) or high (total score: 5 to 9).

### 2.5 Data analysis

Statistical analyses were performed using Stata version 18 (StataCorp. LLC, College Station, USA). We derived pooled estimates of knowledge outcomes by random‐effects meta‐analysis based on the DerSimonian–Laird approach.^18^ We assessed the robustness of our findings in sensitivity analyses that excluded studies with a high risk of bias.

Interstudy heterogeneity was assessed using Cochran's *Q* test, which gives values for chi‐square (*X* ^2^ *)* and corresponding *P‐*value. The percentage of the total variation across studies attributable to heterogeneity was estimated using Higgin's *I* ^*2*^ statistic.^19^ Publication bias was assessed using funnel plots and Egger's regression test.^20^ Subgroup analyses were conducted to determine the pooled estimates of correct knowledge across different world regions and categories of healthcare students.

### 2.6 Nomenclature of targets and ligands

No key protein targets or ligands are reported in this study.

## 3 RESULTS

### 3.1 Study selection and characteristics

**FIGURE 1:**
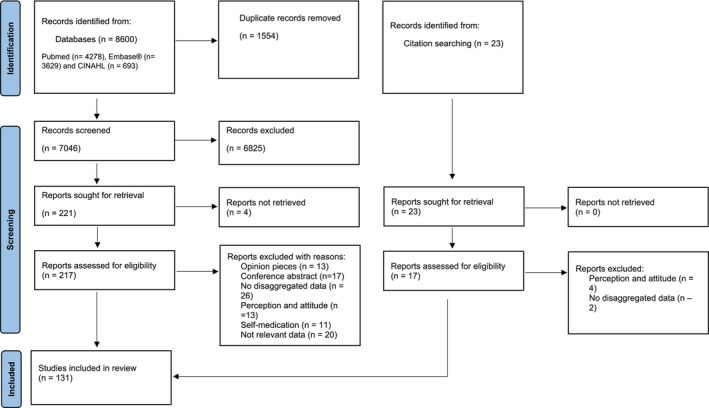
Flow diagram of article selection process.

A total of 8623 articles were identified, comprising 8600 records retrieved from database searches and 23 records obtained through citation tracking. After removing duplicates and irrelevant records, we assessed 234 full‐text articles. Of these, 131 met the inclusion criteria and were included in this systematic review and meta‐analysis (Figure 1). These 131 studies included 56 674 healthcare students. Medical students were the most frequently studied group among healthcare students, with 71 article references (slightly more than half of all included papers). Pharmacy students followed with 44 references, while nursing, dentistry and veterinary students were indicated in 28, 21 and 9 articles, respectively.

The 131 studies included in this review involved participants from 43 countries, with four of these studies incorporating samples from multiple countries. The majority of articles came from South Asia (52), followed by the Middle East and North Africa and Sub‐Saharan Africa, contributing to 24 and 23 studies, respectively. East Asia and the Pacific and Europe and Central Asia contributed to 13 studies each, while Latin America and Caribbean accounted for 4 studies and North America had 1. One study spanned multiple regions (Table S3).

### 3.2 Risk of bias in studies

The total MERSQI scores for the 131 articles included in this review ranged from 7.00 to 14.00, with a mean score of 10.40 ± 1.54. The highest mean domain scores were for data analysis (2.70 ± 0.49) and type of data (2.66 ± 0.75). Objective outcome measures were used in 83.2% (*n* = 109) of the included studies, contributing to the relatively high score in the type of data domain (Table S4). Among these, 49 studies used multiple‐choice formats, 45 used dichotomous (true/false) items and 15 employed mixed strategies. Almost all studies assessed recall of information, with only one incorporating scenario‐based questions. The lowest scores were for study design (1.01 ± 0.08) and validity (0.76 ± 0.79) domains (Table S4).

Nearly all (97.7%) of the studies were single‐group cross‐sectional or posttest‐only designs. Slightly more than a quarter of the studies involved participants from three or more institutions. In addition, 53 (40.5%) of the included studies achieved response rates of 75% or higher. Face validity, either explicitly stated or inferred from item relevance confirmed by appropriate experts, was indicated in 80 articles (61.1%), while content validity, ensuring items covered an appropriate range of topics, was reported in 61 articles (46.6%). However, only 34 (26.0%) of the studies reported on internal structure. Cronbach's *α* was used in 32 studies, while intraclass correlation coefficient (ICC) and Kuder–Richardson 20 (KR20) were each reported in one article.

Overall, the risk of bias was considered low in 14 (10.7%) of the included studies, moderate in 96 (73.3%) and high in 21 (16.0%).

### 3.3 Knowledge of antibiotic use

#### 3.3.1 Bacterial and viral infections

The majority of healthcare students correctly understood that antibiotics are effective against bacterial infections but ineffective against viruses. The pooled estimates were 88.7%, 95% confidence interval: 87.0–90.5 and 70.0% (95% CI: 65.6–74.4), respectively (Table 1). After excluding low‐quality studies, the pooled estimates were 88.0% (95% CI: 85.9–90.1) for correct knowledge of the effectiveness of antibiotics against bacterial infections and 71.3% (95% CI: 66.6–76.0) for correct understanding of the ineffectiveness of antibiotics against viruses. These estimates remain consistent with those prior to the sensitivity analyses.

**TABLE 1:** Pooled estimates of healthcare students' knowledge of antibiotic use.

World region	Antibiotics are effective in treating bacterial infections		Antibiotics are effective against viruses		Antibiotics are effective against cold and flu		Treating the common cold or flu with antibiotics will speed up recovery		Using antibiotics can lead to side effects or risks, including diarrhoea, colitis and allergic reactions	
	*N*	Correct knowledge % (95% CI)	*N*	Correct knowledge % (95% CI)	*N*	Correct knowledge % (95% CI)	*N*	Correct knowledge % (95% CI)	*N*	Correct knowledge % (95% CI)
East Asia and Pacific	7	91.6 (87.5–95.7)	6	58.9 (45.9–71.8)	6	59.1 (39.7–78.6)	3	50.6 (30.4–87.7)	2	81.4 (79.7–83.0)
Europe and Central Asia	8	85.3 (79.0–91.5)	10	80.5 (69.3–91.8)	8	74.0 (64.7–83.3)	4	62.1 (25.7–98.6)	8	80.6 (70.7–90.5)
Latin America and Caribbean	0	ND	2	70.3 (63.6–76.9)	2	59.0 (52.1–65.9)	0	ND	0	ND
Middle East and North Africa	11	87.1 (83.0–91.2)	14	69.0 (61.3–76.8)	7	44.9 (32.0–57.9)	4	59.1 (30.4–87.7)	9	66.8 (52.9–80.7)
North America	0	ND	1	98.0 (94.5–99.3)	1	98.0 (94.5–99.3)	1	98.0 (94.5–99.3)	1	96.0 (91.8–98.1)
South Asia	25	89.8 (87.4–92.2)	34	67.9 (61.5–74.2)	17	57.7 (46.0–69.4)	17	41.3 (33.5–49.1)	16	79.7 (73.2–86.2)
Sub‐Saharan Africa	8	88.4 (82.1–94.7)	7	73.6 (58.3–88.9)	9	52.4 (40.8–64.1)	5	62.5 (44.5–80.6)	8	80.2 (69.5–90.8)
Overall	59	88.7 (87.0–90.5)	74	70.0 (65.6–74.4)	51	57.9 (51.5–64.3)*	34	51.5 (41.6–61.4)	44	77.9 (73.6–82.2)

*Note*: Estimate includes additional studies across multiple regions.

Abbreviation: ND, not determined.

**FIGURE 2:**
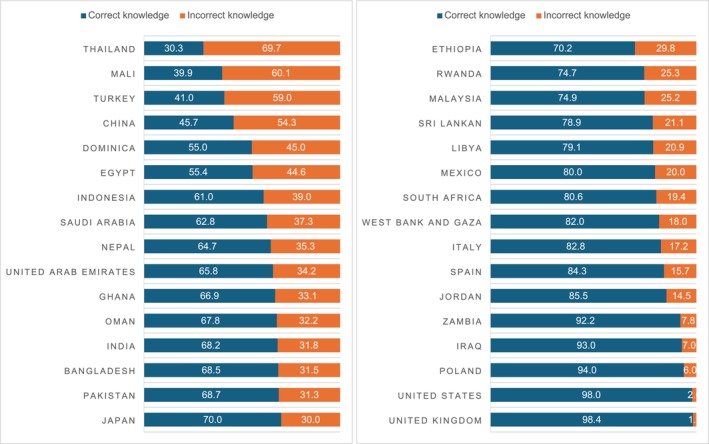
Country level estimates of healthcare students with correct knowledge that antibiotics are ineffective against viruses.

There were no significant variations in the understanding that antibiotics are effective against bacterial infections and ineffective against viruses across world regions, as indicated by the substantial overlap in the 95% confidence intervals (Table 1). However, notable differences were observed in the understanding of the ineffectiveness of antibiotics against viruses at the country level. For example, Thailand (30.3%, 95CI: 23.8–37.8), Mali (39.9%, 95% CI: 35.5–44.5), Turkey (41.0%, 95% CI: 38.7–43.3) and China (45.7%, 95% CI: 43.5–47.9) demonstrated lower levels of knowledge, whereas Poland (94.0%, 95% CI: 90.4–97.5), the United States (98.0%, 95% CI: 94.5–99.3) and the United Kingdom (98.4%, 95% CI: 97.2–99.6) exhibited higher levels of understanding (Figure 2 and Table S5).

#### 3.3.2 Colds and flu

**FIGURE 3:**
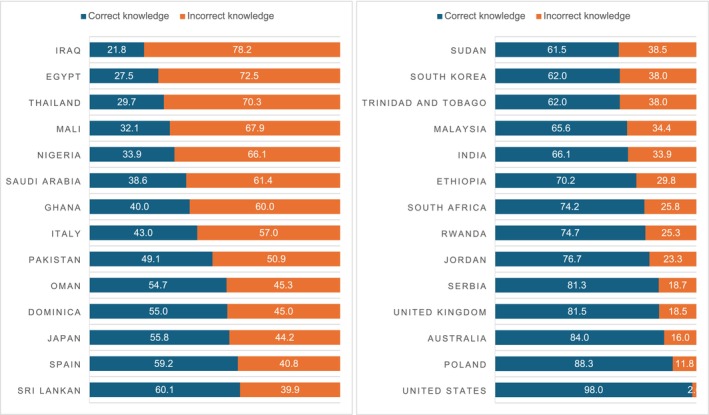
Country level estimates of healthcare students with correct knowledge that antibiotics are ineffective against colds and flu.

Only 57.9% (95% CI: 51.5–64.3) knew that antibiotics are ineffective against colds and flu. Considerable differences were found across countries regarding the understanding that antibiotics are ineffective for colds and flu. Low levels of knowledge were reported among healthcare students in Iraq (21.8%, 95% CI: 15.6–29.6), Egypt (27.5%, 95% CI: 22.1–33.7), Thailand (29.7%, 95% CI: 27.2–32.4), Mali (32.1%, 95% CI: 27.9–36.6) and Nigeria (33.9%, 95% CI: 31.1–36.8). In contrast, higher levels of knowledge were observed in Serbia (81.3%, 95%CI: 77.2–84.8), the United Kingdom (81.5%, 95% CI: 70.9–92.2), Australia (84.0%, 95% CI: 80.6–86.9), Poland (88.3%, 95% CI: 86.4–90.2) and the United States (98.0%, 95% CI: 94.5–99.3) (Figure 3 and Table S6).

Furthermore, just over half (51.5%, 95% CI: 41.6–61.4) of the students from the included studies correctly responded to the question that ‘antibiotics do not speed up recovery from common colds and flu’. There were substantial variations in correct responses to this outcome, across different regions, ranging from 41.3% (95% CI: 33.5–49.1) in South Asia to 98.0% (95% CI: 94.5–99.3) in North America (Table 1). Similarly, we observed a substantial variation in the country‐level estimates of correct response to whether antibiotics speed up recovery from common colds and flu. Low levels of accurate knowledge in this area were observed in several countries, including Sri Lanka (17.7%, 95% CI: 10.8–27.6), Turkey (28.8%, 95% CI: 26.6–31.0), Saudi Arabia (33.5%, 95% CI: 28.3–39.2), United Arab Emirates (34.2%, 95% CI: 30.5–38.1), Ethiopia (35.0%, 95% CI: 30.0–40.4), Pakistan (37.3%, 95% CI: 33.9–40.8) and China (37.5%, 95% CI: 35.9–39.0). On the other hand, healthcare students from Spain (94.5%, 95% CI: 92.3–96.1), the United Kingdom (97.0%, 95% CI: 93.4–98.7) and the United States (98.0%, 95% CI: 94.5–99.3) showed high levels of accurate understanding that antibiotics do not speed up the recovery from the common cold and flu (Table S7).

Eleven studies assessed the proportion of healthcare students who correctly understood that colds and flu are not caused by bacteria. The pooled estimate of correct knowledge across these studies was 57.4% (95% CI: 49.0–65.8). These studies were conducted in four regions: South Asia, the Middle East and North Africa, Latin America and the Caribbean and Sub‐Saharan Africa. The pooled regional estimates were 55.3% (95% CI: 35.6–74.9), 58.7% (95% CI: 50.1–67.3), 59.6% (95% CI: 52.7–66.5) and 60.9% (95% CI: 53.7–67.7), respectively.

### 3.4 Knowledge of antibiotic resistance

We estimated that most healthcare students (89.8%, 95% CI: 88.0–91.7) are aware of antibiotic resistance. Awareness levels ranged from 86.9% (95% CI: 82.5–91.3) in Middle East and North Africa to 93.0% (95% CI: 88.1–96.0) in North America. Additionally, a significant majority (84.2%, 95% CI: 81.1–87.2) recognized antibiotic resistance as a global public health threat. However, only 62.2% (95% CI: 52.4–72.0) knew that antibiotic‐resistant bacteria can spread from person to person (Table 2).

**TABLE 2:** Pooled estimates of healthcare students' knowledge of antibiotic resistance outcomes.

World region	Using broad‐spectrum antibiotics instead of equally effective narrow‐spectrum options contributes to the development of antibiotic resistance		The unnecessary use of antibiotics makes them ineffective or leads to resistance		Patients should stop taking their prescribed antibiotics as soon as they feel better		Resistant infections could make medical procedures like surgery, organ transplants and cancer treatment much more difficult		Antibiotic‐resistant bacteria can spread from person to person	
	*N*	Correct knowledge % (95% CI)	*N*	Correct knowledge % (95% CI)	*N*	Correct knowledge % (95% CI)	*N*	Correct knowledge % (95% CI)	*N*	Correct knowledge % (95% CI)
East Asia and Pacific	1	79.2 (74.6–83.1)	0	ND	4	85.1 (76.5–93.7)	4	83.1 (70.4–95.8)	4	66.9 (49.0–84.7)
Europe and Central Asia	1	88.0 (82.5–91.9)	10	90.8 (87.0–94.5)	6	84.3 (78.1–90.4)	0	ND	0	ND
Latin America and Caribbean	1	74.5 (71.6–78.9)	1	88.0 (79.4–93.3)	2	88.0 (85.4–90.5)	0	ND	0	ND
Middle East and North Africa	3	70.2 (54.3–86.1)	14	83.9 (78.9–88.0)	9	71.8 (59.6–84.1)	1	65.8 (59.9–71.3)	4	59.5 (43.6–75.4)
North America	1	84.0 (77.6–88.8)	1	98.0 (94.5–99.3)	1	96.0 (91.8–98.1)	0	ND	0	ND
South Asia	6	69.2 (58.8–79.6)	26	86.7 (83.5–89.9)	12	65.8 (55.0–76.6)	2	70.8 (67.7–73.8)	1	59.2 (54.7–63.6)
Sub‐Saharan Africa	7	67.8 (59.4–78.2)	11	87.2 (82.3–92.1)	7	82.8 (74.7–90.8)	2	72.8 (68.0–77.7)	1	57.3 (49.5–64.8)
Overall	20	71.7 (66.6–76.9)	63	87.0 (85.1–89.0)	42	76.2 (72.2–80.2)	9	76.1 (67.0–85.3)	10	62.2 (52.4–72.0)

*Note*: Estimate includes additional studies across multiple regions.

Abbreviation: ND, not determined.

Nearly three‐quarters of healthcare students knew that using broad‐spectrum antibiotics instead of equally effective narrow‐spectrum options contributes to antibiotic resistance. Also, just over three‐quarters understood that resistant infections could complicate medical procedures like surgery, organ transplants and cancer treatments. Pooled estimates for these understandings were 71.7% (95% CI: 66.6–76.9) and 76.1% (95% CI: 67.0–85.3), respectively. Additionally, students demonstrated a high level of understanding in areas related to whether antibiotics should be stopped once patients feel better and whether unnecessary use makes them ineffective or leads to resistance (Table 2).

The subgroup analyses of healthcare student categories indicated no notable variation, as the 95% confidence intervals overlapped substantially in all the outcomes investigated (Table 3).

**TABLE 3:** Pooled estimates of healthcare students' knowledge of antibiotic use and resistance by professional categories,

Knowledge area	Medical students		Pharmacy students		Nursing students		Veterinary students	
	*N*	Correct knowledge % (95% CI)	*N*	Correct knowledge % (95% CI)	*N*	Correct knowledge % (95% CI)	*N*	Correct knowledge % (95% CI)
Antibiotics are effective in treating bacterial infections	23	90.8 (88.5–93.2)	7	90.6 (85.8–95.4)	3	89.7 (80.5–99.0)	3	93.0 (88.7–97.2)
Antibiotics are effective against viruses.	29	75.9 (70.9–80.9)	6	68.1 (51.9–84.3)	6	71.6 (50.0–93.2)	5	75.8 (58.2–93.3)
Antibiotics are effective against cold and flu.	14	67.0 (56.5–77.4)	14	52.2 (42.8–61.6)	9	58.9 (38.0–79.8)	3	62.7 (44.3–81.1)
Treating the common cold or flu with antibiotics will speed up recovery	10	56.2 (45.1–67.2)	8	53.2 (30.2–76.3)	3	74.2 (31.2–117.1)	1	56.8 (54.2–59.3)
Bacteria cause the common cold and flu	5	58.7 (41.2–76.1)	6	56.1 (47.0–65.3)		ND		ND
Using antibiotics can lead to side effects or risks, including diarrhoea, colitis, and allergic reactions	12	78.1 (70.6–85.7)	7	82.7 (75.6–89.8)	8	73.6 (58.6–88.7)	2	65.5 (61.8–69.2)
Using broad‐spectrum antibiotics instead of equally effective narrow‐spectrum options contributes to the development of antibiotic resistance.	9	71.5 (62.4–80.6)	5	76.3 (67.9–84.6)	3	62.8 (36.6–89.0)	0	ND
The unnecessary use of antibiotics makes them ineffective or leads to resistance	20	88.3 (84.3–92.4)	13	86.6 (82.9–90.3)	4	80.6 (68.0–93.3)	4	93.3 (88.6–97.9)
Patients should stop taking their prescribed antibiotics as soon as they feel better	14	76.0 (69.3–82.6)	11	71.4 (60.2–82.5)	5	83.1 (74.5–91.6)	2	76.8 (73.1–80.4)
Resistant infections could make medical procedures like surgery, organ transplants and cancer treatment much more difficult	0	ND	4	82.2 (68.3–96.1)	2	74.9 (72.8–77.1)	0	ND
Antibiotic‐resistant bacteria can spread from person to person.	1	59.2 (54.0–64.2)	3	72.6 (52.4–93.0)	1	60.8 (58.0–63.6)	0	ND

Abbreviation: ND, not determined.

Generally, significant heterogeneities were observed across the included studies for all the investigated knowledge outcomes (Table S8). For instance, meta‐analyses yielded *I* ^2^ values of 97.7% ( $X^{2}$: 2430.0; *P* < 0.001) and 99.2% ( $X^{2}$: 8430.9.5; *P* < 0.001), for outcomes assessing the correct knowledge of antibiotic effectiveness against bacterial infections and the understanding of antibiotic ineffectiveness against viruses, respectively. The Egger test results identified significant publication bias in seven of the eleven outcomes analysed (Table S8).

## 4 DISCUSSION

Our systematic review and meta‐analysis indicate that healthcare students generally demonstrate a substantial level of antibiotic knowledge across many of the evaluated outcomes. We found that students possess a good understanding that antibiotics are effective against bacterial infections but ineffective against viruses. However, only 57.9% (95% CI: 51.5–64.3) knew that antibiotics are ineffective against colds and flu. Furthermore, just over half (51.5%, 95% CI: 41.6–61.4) of the students correctly reported that antibiotics do not speed up recovery from common colds and flu. These knowledge gaps, particularly regarding misunderstandings about common colds and flu, have substantial population health implications for the rational use of antibiotics and the prevention of resistance.

Firstly, the misconception that antibiotics are effective against colds and flu can lead to misuse, overprescribing or inappropriate supply, thereby contributing to antibiotic resistance.^21^ This resistance poses a serious threat to global public health, making infections harder to treat and leading to increased morbidity and mortality.^3^, ^4^ Secondly, healthcare students who lack adequate knowledge might prescribe or supply antibiotics unnecessarily once they qualify.^10^ This practice not only fails to treat the underlying viral infections but also exposes patients to unnecessary side effects and potential complications.^22^, ^23^ Thirdly, these knowledge gaps can undermine strategies aimed at preventing antibiotic resistance, including antibiotic stewardship programmes. Additionally, as future first points of contact for patients seeking medical advice, healthcare students' understanding of antibiotics directly impacts their ability to effectively educate the public and promote rational antibiotic use. This understanding, in turn, influences overall public awareness and behaviour regarding antibiotic consumption. Our findings underscore the need for enhanced education and training in medical and healthcare curricula. Emphasizing the nature of viral infections and the inappropriateness of using antibiotics to treat or expedite the recovery of these infections can better equip future healthcare professionals to make informed decisions and accurately educate their patients.^1^, ^23^ The knowledge gaps identified by this systematic review also have implications for current practitioners, as many of the healthcare students included in the reviewed studies are now in practice. Therefore, there is a need for ongoing education and training beyond initial qualifications. Healthcare institutions might implement mandatory continuing education courses on the latest developments in antibiotic use and stewardship practices, ensuring that healthcare professionals remain updated on best practices throughout their careers. These interventions are particularly crucial in countries such as Iraq, Thailand, Egypt and Nigeria where studies have reported the widespread non‐prescription supply of antibiotics and their use for upper respiratory tract infections,^1^, ^24^, ^25^, ^26^, ^27^ and our review revealed a limited understanding of the ineffectiveness of antibiotics in treating the common cold and flu in these nations.

The majority of healthcare students included in the studies we reviewed are aware of antibiotic resistance and recognize it as a global public health threat. These findings suggest that current educational and awareness programmes are effective in increasing knowledge. Although awareness does not always translate into practice, these well‐informed students will be better equipped to educate their patients once they qualify. This can lead to more informed patients who understand the importance of adhering to prescribed antibiotic regimens, the dangers of self‐medication and the necessity of avoiding unnecessary antibiotic use. Increased awareness among healthcare students can also influence future healthcare policies. As these students enter the workforce and potentially assume leadership roles, their understanding of antibiotic resistance can drive policies that support the development of new antibiotics, regulate antibiotic use and promote research into alternative treatments. Antibiotic resistance is a global issue requiring a coordinated international response across multiple countries and sectors.^28^, ^29^ Our review findings, showing widespread knowledge of antibiotic resistance across all world regions, indicate that future generations of healthcare professionals are prepared to contribute to global efforts in combating antibiotic resistance.

This is the first systematic review to provide a comprehensive current evaluation of healthcare students' knowledge of antibiotic use and resistance. It highlights both strengths and weaknesses that should be addressed in curricula and training programmes. The strength of our review is in its extensive coverage involving data from 56 674 students across 43 countries. This review, however, has some limitations; therefore, the findings should be interpreted with caution. Only 14 (10.7%) of the 131 included studies were deemed to have a low risk of bias, indicating high methodological quality. Furthermore, publication bias was evident in the majority of the outcomes studied. The knowledge outcomes assessed in this review primarily relate to general understanding of antibiotic use and common misconceptions, rather than detailed aspects of prescribing; as such, the findings may not fully reflect healthcare students' preparedness for prescribing practices or their future clinical competence in antibiotic use. The significant heterogeneity observed across studies may have influenced our pooled estimates,^30^ and is likely attributable to differences in study populations, educational settings, cognitive level, assessment format and the scope of content assessed. Such heterogeneity is not uncommon in meta‐analyses of studies assessing knowledge of antibiotics and supports our use of a random‐effects meta‐analysis model, which accounts for variability between studies.^31^, ^32^ However, this variability should be considered when interpreting the pooled findings, as differences in how knowledge was assessed may limit comparability across studies. Importantly, the sensitivity analyses carried out by excluding low‐quality studies revealed comparable estimates, providing further reassurance of the robustness of our findings.

Many of the reviewed studies lacked information or clarity regarding the content validity of the instruments or questions used to assess antibiotic knowledge. Describing the chosen validity approaches, including the face validity of items and the content validity of tests, would greatly enhance articles evaluating healthcare students' knowledge of antibiotics. Additionally, the reliability of the study instruments, while extremely valuable, was not always determined or reported. Furthermore, studies that reported the internal consistency of their instruments using Cronbach's *α* often did not include the standard error of measurement, which provides insights into the precision of the measurement instruments.^33^ Reporting the reliability of instruments in a study is crucial for the scientific rigour and credibility of the research. When assessing knowledge, it is essential that the instrument yields stable and consistent results. Reporting reliability provides confidence that the results are dependable and not influenced by random errors.^33^, ^34^ Moreover, reporting the reliability of knowledge‐assessment instruments allows other researchers to replicate the study and compare results across different studies. This replication is fundamental to scientific progress, enabling researchers to verify findings and build on existing knowledge. Additionally, comprehensive reporting of the reliability of the instruments allows for the identification and refinement of items within the questionnaires that show low reliability. This enhancement of the overall reliability of the instrument, when adopted in other studies, ultimately improves its quality and precision over time.

Our review included single or limited reports from some countries and regions, and many reports concerned studies conducted in a single institution, which were not nationally representative of the institutions in the study countries. These factors may affect the generalisability of our findings. Additionally, it is possible that the antibiotic knowledge levels of students from these countries may differ from what we found. Our search strategy excluded articles that were not reported in English, potentially missing important studies published in non‐English languages. Moreover, medical, pharmacy and nursing students represented the majority of the population groups studied in the papers we reviewed. Therefore, our findings may more accurately reflect the situation within medicine, pharmacy and nursing, as other disciplines are currently under‐researched or under‐reported. Additionally, many of the studies conducted among dental students were excluded because they investigated dentistry‐specific outcomes that were not covered in our study. Nonetheless, our study offers valuable insights into healthcare students' understanding of antibiotic use and resistance, which could drive the development of curricular interventions.

## 5 CONCLUSION

Healthcare students demonstrated a substantial level of antibiotic knowledge across many of the outcomes evaluated in this review. They showed a strong understanding that antibiotics are effective against bacterial infections but not against viruses. However, a notable knowledge gap was identified in their understanding that antibiotics are ineffective for treating colds and flu. Additionally, many students were unaware that antibiotics do not accelerate recovery from common colds and flu. Addressing these knowledge gaps through targeted education and training that underscore the nature of viral infections and the ineffectiveness of antibiotics in treating or accelerating recovery from such infections is vital. This strategy has critical public health implications, promoting the rational use of antibiotics and combating the growing issue of antibiotic resistance.

## AUTHOR CONTRIBUTIONS

Asa Auta led the study design, with contributions from Erick Wesley Hedima and Barry Strickland‐Hodge. Asa Auta and Emmanuel O. Adewuyi performed the literature searches. Asa Auta and Erick Wesley Hedima screened the retrieved articles. Asa Auta extracted the data from included studies. Emmanuel O. Adewuyi, Shalkur David and Enoche Florence Oga cross‐checked the data extraction. Asa Auta performed the quality assessment of studies, which was verified by Emmanuel Agada David, Lomikga Balachandran and Shalkur David. Asa Auta conducted the statistical analyses, which were verified by Davies Adeloye. Asa Auta wrote the initial draft, and Barry Strickland‐Hodge reviewed it. All authors critically reviewed subsequent drafts and read and approved the submitted version.

## CONFLICT OF INTEREST STATEMENT

The authors declare that they have no conflicts of interest.

## Supporting information


**Table S1:** Search strategy.
**Table S2**: knowledge outcomes evaluated.
**Table S3**: Characteristics of included studies.
**Table S4**: MERSQI domain and items cores for included studies.
**Table S5**: Country level estimates of healthcare students with correct knowledge that antibiotics are ineffective against viruses.
**Table S6**: Country level estimates of healthcare students with correct knowledge that antibiotics are ineffective against colds and flu.
**Table S7**: Country level estimates of healthcare students with correct knowledge that antibiotics that antibiotics do not speed up the recovery from the common cold and flu.
**Table S8**: Heterogeneity and Egger's bias estimates across studies.

## Data Availability

The data that support the findings of this study are available from the corresponding author upon reasonable request.
