# Supplementary material for: Global and regional knowledge of antibiotic use and resistance among healthcare students: A systematic review and meta‐analysis
Source: Br J Clin Pharmacol. 2026 Apr 23;92(7):1994–2004. doi: 10.1002/bcp.70575 (PMC13304241; doi:10.1002/bcp.70575)
Supplement: Supplementary file 1 — Table S1: Search strategy. Table S2: knowledge outcomes evaluated. Table S3: Characteristics of included studies. Table S4: MERSQI domain and items cores for included studies. Table S5: Country level estimates of healthcare students with correct knowledge that antibiotics are ineffective against viruses. Table S6: Country level estimates of healthcare students with correct knowledge that antibiotics are ineffective against colds and flu. Table S7: Country level estimates of healthcare students with correct knowledge that antibiotics that antibiotics do not speed up the recovery from the common cold and flu. Table S8: Heterogeneity and Egger's bias estimates across studies. [file BCP-92-1994-s001.docx]

# Supplementary information

## Supplementary Table 1: Search strategy

Pubmed

| **#** | **Searches** |
| --- | --- |
| 1 | Medical OR Pharmacy [MeSH Terms] OR Pharmacy OR Dent* OR Nurs* OR physiotherapy OR Allied Health OR Health OR Health [MeSH Terms] OR Healthcare OR Midwifery OR midwifery [MeSH Terms] OR Radiograph* OR Biomedical OR Optometry [MeSH Terms] OR Optometry OR Veterinary OR Animal health |
| 2 | Students [MeSH Terms] OR Students OR Education [MeSH Terms] OR Education OR Undergraduate* |
| 3 | Antibiotic* OR antimicrobial* OR anti bacterial agents [MeSH Terms] OR antibacterial OR drug resistance, microbial [MeSH Terms] OR antibiotic resistance OR antimicrobial resistance |
| 4 | Knowledge [MeSH Terms] OR knowledge OR competenc* OR skill* |
| 5 | 1 AND 2 AND 3 AND 4 |
| 6 | limit 5 to yr="2015 - 2025" |

Embase and CINAHL

| **#** | **Searches** |
| --- | --- |
| 1 | Medical OR Pharmacy OR Dent* OR Nurs* OR physiotherapy OR Allied Health OR Health OR Healthcare OR Midwifery OR Radiograph* OR Biomedical OR Optometry OR Veterinary OR Animal health |
| 2 | Students OR education OR undergraduate* |
| 3 | Antibiotic* OR antimicrobial* OR antibacterial OR antibiotic resistance OR antimicrobial resistance |
| 4 | Knowledge OR competenc* OR skill* |
| 5 | 1 AND 2 AND 3 AND 4 |
| 6 | limit 5 to yr="2015 - 2025" |

## Supplementary Table 2: knowledge outcomes evaluated

| Question | Correct response |
| --- | --- |
| Antibiotics are effective in treating bacterial infections. | True |
| Antibiotics are effective against viruses. | False |
| Antibiotics are effective against cold and flu. | False |
| Treating the common cold or flu with antibiotics will speed up recovery. | False |
| Cold and flu are caused by bacteria. | False |
| Using antibiotics can lead to side effects or risks, including diarrhoea, colitis, and allergic reactions. | True |
| Patients should stop taking their prescribed/recommended antibiotics as soon as they feel better. | False |
| Using broad-spectrum antibiotics instead of equally effective narrow-spectrum options contributes to the development of antibiotic resistance. | True |
| The unnecessary use of antibiotics makes them ineffective or leads to resistance. | True |
| Resistant infections could make medical procedures like surgery, organ transplants and cancer treatment much more difficult. | True |
| Antibiotic-resistant bacteria can spread from person to person. | True |

## Supplementary Table 3: Characteristics of included studies

| **Study** | **Year** | **Country** | **Region** | **Study design** | **Sample size** | **Student group** | **Number of institutions** | **Outcome indicators** | **Risk of bias** |
| --- | --- | --- | --- | --- | --- | --- | --- | --- | --- |
| Abdelkarim et al^1^ | 2024 | Sudan | Sub-Saharan Africa | Cross-sectional | 109 | ② | Multiple | µ⮿ | Moderate |
| Abdu-Aguye et al^2^ | 2022 | Nigeria | Sub-Saharan Africa | Cross-sectional | 164 | ② | Multiple | µ | Moderate |
| Abuawad et al^3^ | 2024 | West Bank and Gaza | Middle East & North Africa | Cross-sectional | 384 | ➀ | Multiple | µ | Moderate |
| Abubakar et al^4^ | 2020 | Multiple countries | Multiple | Cross-sectional | 211 | ② | Multiple | µ⮿ | Low |
| Ahmad et al^5^ | 2015 | Trinidad and Tobago | Latin America & Caribbean | Cross-sectional | 108 | ② | Single | ⮿ | Moderate |
| Ahmad et al^6^ | 2015 | India | South Asia | Cross-sectional | 137 | ② | Multiple |  | Moderate |
| Akande-Sholabi and Ajamu^7^ | 2021 | Nigeria | Sub-Saharan Africa | Cross-sectional | 866 | ➀②③ | Single |  | Moderate |
| Akbar et al^8^ | 2021 | Saudi Arabia | Middle East & North Africa | Cross-sectional | 284 | ②③⑦ | Single | µ⮿ | Moderate |
| Al-Qerem et al^9^ | 2022 | Jordan | Middle East & North Africa | Cross-sectional | 890 | ② | Multiple | ⮿ | Moderate |
| Al-Sadoon^10^ | 2020 | Iraq | Middle East & North Africa | Cross-sectional | 209 | ➀ | Single |  | High |
| Alsaleh et al^11^ | 2020 | Saudi Arabia | Middle East & North Africa | Cross-sectional | 503 | ➀➄ | Multiple | µ | Moderate |
| Alsayed et al^12^ | 2022 | Multiple countries | Middle East & North Africa | Cross-sectional | 2322 | ⑦ | Multiple | ⮿ | Moderate |
| Al-Taani et al^13^ | 2022 | Jordan | Middle East & North Africa | Cross-sectional | 716 | ➀②③ | Multiple | µ | Moderate |
| Assar et al^14^ | 2020 | Egypt | Middle East & North Africa | Cross-sectional | 963 | ➀ | Multiple | ⮿ | Moderate |
| Augie et al^15^ | 2021 | Multiple countries | Sub-Saharan Africa | Cross-sectional | 276 | ➀ | Multiple |  | Moderate |
| Azechi et al^16^ | 2022 | Japan | East Asia & Pacific | Cross-sectional | 330 | ② | Single |  | Moderate |
| Bharti et al^17^ | 2020 | India | South Asia | Cross-sectional | 359 | ➀③➄ | Single |  | Moderate |
| Bonna et al^18^ | 2024 | Bangladesh | South Asia | Cross-sectional | 501 | ➀ | Multiple |  | Low |
| Bukhsh et al^19^ | 2019 | Pakistan | South Asia | Cross-sectional | 399 | ➀②➄ | Multiple |  | Moderate |
| Chakravarty et al^20^ | 2022 | India | South Asia | Cross-sectional | 1505 | ➀ | Multiple |  | Moderate |
| Choi et al^21^ | 2019 | South Korea | East Asia & Pacific | Cross-sectional | 295 | ③ | Single |  | Low |
| Elmahi et al^22^ | 2022 | Sudan | Sub-Saharan Africa | Cross-sectional | 1110 | ➀ | Multiple |  | High |
| Fetensa et al^23^ | 2020 | Ethiopia | Sub-Saharan Africa | Cross-sectional | 232 | ②③⑦ | Single |  | High |
| Gayathri et al^24^ | 2021 | India | South Asia | Cross-sectional | 202 | ➄ | Single | ⮿ | Moderate |
| Hagiya et al^25^ | 2020 | Japan | East Asia & Pacific | Cross-sectional | 661 | ➀ | Single |  | Moderate |
| Haque et al^26^ | 2019 | Malaysia | East Asia & Pacific | Cross-sectional | 204 | ➀ | Single | µ | Moderate |
| Harakeh et al^27^ | 2015 | Saudi Arabia | Middle East & North Africa | Cross-sectional | 1042 | ➀ | Multiple |  | High |
| Hayat et al^28^ | 2021 | Pakistan | South Asia | Cross-sectional | 296 | ② | Multiple | µ | Low |
| Hayat et al^29^ | 2022 | Pakistan | South Asia | Cross-sectional | 411 | ➀ | Multiple | µ | Low |
| Higuita-Gutiérrez et al^30^ | 2020 | Columbia | Latin America & Caribbean | Cross-sectional | 532 | ➀ | Multiple | ⮿ | Moderate |
| Hu et al^31^ | 2018 | China | East Asia & Pacific | Cross-sectional | 1819 | ➀ | Multiple |  | Moderate |
| Hussain et al^32^ | 2021 | Pakistan | South Asia | Cross-sectional | 496 | ②⑥ | Single |  | Moderate |
| Hussain et al^33^ | 2021 | Pakistan | South Asia | Pre-post study | 500 | ➀⑦ | Single | ⮿ | Moderate |
| Inacio et al^34^ | 2017 | United Kingdom | Europe & Central Asia | Cross-sectional | 185 | ② | Single | µ | Moderate |
| Jairoun et al^35^ | 2019 | United Arab Emirates | Middle East & North Africa | Cross-sectional | 600 | ⑦ | Single | µ | Moderate |
| Jayawardhana et al^36^ | 2023 | Sri Lankan | South Asia | Cross-sectional | 347 | ➀ | Single |  | High |
| Jayaweerasingham et al^37^ | 2019 | Sri Lankan | South Asia | Cross-sectional | 199 | ③ | Single | µ | Moderate |
| Jianvitayakij et al^38^ | 2024 | Thailand | East Asia & Pacific | Cross-sectional | 1180 | ③ | Multiple | µ | Low |
| Kandasamy et al^39^ | 2020 | Saudi Arabia | Middle East & North Africa | Cross-sectional | 220 | ② | Single | ⮿ | Moderate |
| Kanyike et al^40^ | 2022 | Uganda | Sub-Saharan Africa | Cross-sectional | 681 | ➀②③➄⑦ | Multiple |  | Moderate |
| Karthik et al^41^ | 2019 | India | South Asia | Cross-sectional | 300 | ② | Single | ⮿ | High |
| Keziah and Pandunrangan^42^ | 2020 | India | South Asia | Cross-sectional | 1127 | ➄⑦ | Single | ⮿ | Moderate |
| Khajuria et al^43^ | 2019 | India | South Asia | Cross-sectional | 140 | ➀ | Single | µ | High |
| Khan et al^44^ | 2021 | Pakistan | South Asia | Mixed methods | 414 | ② | Multiple | ⮿ | Moderate |
| Khanikar et al^45^ | 2024 | India | South Asia | Cross-sectional | 268 | ➀ | Single | µ | Moderate |
| Lubwama et al^46^ | 2021 | Multiple countries | Sub-Saharan Africa | Cross-sectional | 328 | ➀② | Multiple |  | Moderate |
| Manning et al^47^ | 2022 | United States | North America | Pre-post study | 165 | ③ | Single | µ | Moderate |
| Mech et al^48^ | 2023 | India | South Asia | Cross-sectional | 136 | ➀ | Single |  | High |
| Meher et al^49^ | 2020 | India | South Asia | Cross-sectional | 197 | ➀ | Single |  | Low |
| Mohan et al^50^ | 2024 | India | South Asia | Cross-sectional | 150 | ➀ | Single | ⮿ | High |
| Mubarak et al^51^ | 2024 | Saudi Arabia | Middle East & North Africa | Cross-sectional | 131 | ➄ | Multiple |  | Moderate |
| Nisabwe et al^52^ | 2020 | Rwanda | Sub-Saharan Africa | Cross-sectional | 229 | ➀②➄ | Single |  | High |
| Nowbuth et al^53^ | 2023 | Zambia | Sub-Saharan Africa | Cross-sectional | 180 | ➀ | Multiple |  | Moderate |
| Nukaly et al^54^ | 2024 | Saudi Arabia | Middle East & North Africa | Cross-sectional | 353 | ➀ | Single | µ | Moderate |
| Owusu-Ofori et al^55^ | 2021 | Ghana | Sub-Saharan Africa | Cross-sectional | 264 | ③④⑦ | Single | µ | Moderate |
| Pradhan et al^56^ | 2024 | India | South Asia | Cross-sectional | 149 | ② | Single |  | Moderate |
| Precha et al^57^ | 2024 | Thailand | East Asia & Pacific | Cross-sectional | 162 | ➀②③⑦ | Single |  | Moderate |
| Rabano-Blanko et al^58^ | 2019 | Spain | Europe & Central Asia | Cross-sectional | 578 | ③ | Single | µ | Moderate |
| Raees et al^59^ | 2023 | Pakistan | South Asia | Cross-sectional | 1251 | ②③ | Multiple | µ⮿ | Low |
| Rajiah et al^60^ | 2015 | Malaysia | East Asia & Pacific | Cross-sectional | 346 | ② | Multiple |  | Moderate |
| Reena and Ittyachen^61^ | 2022 | India | South Asia | Cross-sectional | 354 | ➀ | Single | ⮿ | Moderate |
| Saikia et al^62^ | 2024 | India | South Asia | Cross-sectional | 97 | ➀ | Single | ⮿ | Moderate |
| Sakeena et al^63^ | 2018 | Sri Lankan | South Asia | Cross-sectional | 466 | ② | Multiple | µ | Low |
| Sakeena et al^64^ | 2021 | Australia | East Asia & Pacific | Cross-sectional | 525 | ② | Multiple | µ | Moderate |
| Sannathimmappa et al^65^ | 2021 | India | South Asia | Cross-sectional | 125 | ➀ | Single | µ | Moderate |
| Scaioli et al^66^ | 2015 | Italy | Europe & Central Asia | Cross-sectional | 1050 | ➀ | Single | µ | Moderate |
| Seid and Hussen^67^ | 2018 | Ethiopia | Sub-Saharan Africa | Cross-sectional | 323 | ②③⑦ | Single |  | Moderate |
| Shah et al^68^ | 2019 | Nepal | South Asia | Cross-sectional | 609 | ➀③➄ | Single | µ | Moderate |
| Shahnaz et al^69^ | 2020 | Pakistan | South Asia | Cross-sectional | 460 | ② | Single | ⮿ | Moderate |
| Sharma et al^70^ | 2022 | India | South Asia | Cross-sectional | 255 | ➀ | Single | µ | High |
| Shrestha^71^ | 2019 | Nepal | South Asia | Cross-sectional | 228 | ➀➄ | Single | µ⮿ | High |
| Sowmya et al^72^ | 2024 | India | South Asia | Cross-sectional | 150 | ➀ | Single | ⮿ | Moderate |
| Struzycka et al^73^ | 2019 | Poland | Europe & Central Asia | Cross-sectional | 752 | ➄ | Multiple |  | Low |
| Tariq et al^74^ | 2021 | Pakistan | South Asia | Cross-sectional | 396 | ➄ | Multiple | ⮿ | Low |
| Tayyab et al^75^ | 2017 | Pakistan | South Asia | Cross-sectional | 223 | ➀ | Single | ⮿ | High |
| Teague et al^76^ | 2023 | South Africa | Sub-Saharan Africa | Cross-sectional | 124 | ③ | Multiple |  | Moderate |
| Yadav et al^77^ | 2024 | India | South Asia | Cross-sectional | 148 | ➀ | Single | ⮿ | Moderate |
| Yahya et al^78^ | 2021 | Iraq | Middle East & North Africa | Cross-sectional | 101 | ② | Multiple | ⮿ | Moderate |
| Yang et al^79^ | 2024 | China | East Asia & Pacific | Cross-sectional | 1959 | ③ | Multiple |  | Moderate |
| Zawahir et al^80^ | 2017 | Sri Lankan | South Asia | Cross-sectional | 79 | ② | Multiple | µ | Moderate |
| Chen et al^81^ | 2021 | Mali | Sub-Saharan Africa | Cross-sectional | 446 | ➀ | Multiple |  | Moderate |
| Golding et al^82^ | 2022 | United Kingdom | Europe & Central Asia | Cross-sectional | 566 | ⑥ | Multiple | µ⮿ | Low |
| Betancourt et al^83^ | 2020 | Mexico | Latin America & Caribbean | Cross-sectional | 86 | ⑥ | Multiple |  | High |
| Chapot et al ^84^ | 2021 | Bangladesh | South Asia | Cross-sectional | 1428 | ⑥ | Multiple | µ | Moderate |
| Almadah et al^85^ | 2024 | Libya | Middle East & North Africa | Cross-sectional | 110 | ➀ | Single |  | Moderate |
| Corrente et al^86^ | 2021 | Italy | Europe & Central Asia | Cross-sectional | 106 | ⑥ | Single |  | Moderate |
| Dass et al^87^ | 2019 | India | South Asia | Cross-sectional | 250 | ➀➄ | Single | µ | Moderate |
| De and Mukherjee^88^ | 2020 | India | South Asia | Cross-sectional | 151 | ➀ | Single |  | Moderate |
| Doe et al^89^ | 2023 | Ghana | Sub-Saharan Africa | Cross-sectional | 1027 | ②③⑦ | Single | µ | Moderate |
| Dyar et al^90^ | 2018 | United Kingdom | Europe & Central Asia | Cross-sectional | 255 | ➀③➄⑥ | Multiple | µ | Moderate |
| Emera et al^91^ | 2024 | Egypt | Middle East & North Africa | Cross-sectional | 1250 | ➀②③➄ | Multiple |  | Low |
| Gupta et al^92^ | 2017 | India | South Asia | Cross-sectional | 132 | ➀ | Single | µ⮿ | Moderate |
| Hameed et al^93^ | 2024 | Iraq | Middle East & North Africa | Cross-sectional | 133 | ➀③⑦ | Single | ⮿ | Moderate |
| Horvat et al^94^ | 2022 | Serbia | Europe & Central Asia | Cross-sectional | 400 | ➀➄⑥ | Single |  | Moderate |
| Kanneppady et al^95^ | 2019 | Malaysia | East Asia & Pacific | Cross-sectional | 260 | ➀ | Single | µ | Moderate |
| Lomi et al^96^ | 2019 | India | South Asia | Cross-sectional | 70 | ➄ | Single |  | Moderate |
| Magbol et al^97^ | 2024 | Sudan | Sub-Saharan Africa | Cross-sectional | 285 | ➀ | Single | µ | High |
| Marta-Costa et al^98^ | 2021 | Portugal | Europe & Central Asia | Cross-sectional | 164 | ➀⑥ | Multiple |  | Moderate |
| Memon et al^99^ | 2021 | Saudi Arabia | Middle East & North Africa | Cross-sectional | 423 | ⑦ | Single |  | Moderate |
| Mudenda et al^100^ | 2022 | Zambia | Sub-Saharan Africa | Cross-sectional | 172 | ② | Single |  | High |
| Nair et al^101^ | 2020 | India | South Asia | Cross-sectional | 246 | ② | Single | ⮿ | High |
| Nayak et al^102^ | 2016 | Nepal | South Asia | Cross-sectional | 176 | ③➄ | Single | ⮿ | High |
| Nwafia et al^103^ | 2024 | Nigeria | Sub-Saharan Africa | Cross-sectional | 550 | ➀ | Single | µ | Moderate |
| Olatunde and Owolabi^104^ | 2022 | Dominica | Latin America & Caribbean | Cross-sectional | 85 | ➀ | Single |  | Moderate |
| Orhan et al^105^ | 2022 | Turkey | Europe & Central Asia | Cross-sectional | 1006 | ③④⑦ | Single | ⮿ | Moderate |
| Panthi et al^106^ | 2020 | Nepal | South Asia | Cross-sectional | 253 | ➀➄ | Single |  | High |
| Sakr et al^107^ | 2020 | Lebanon | Middle East & North Africa | Cross-sectional | 477 | ②⑦ | Single | µ⮿ | Moderate |
| Zulu et al^108^ | 2020 | Zambia | Sub-Saharan Africa | Cross-sectional | 260 | ➀ | Single | µ | Moderate |
| Tarun and Sonal^109^ | 2024 | India | South Asia | Cross-sectional | 400 | ➀➄ | Single | µ | Moderate |
| Syahniar et al^110^ | 2024 | Indonesia | East Asia & Pacific | Cross-sectional | 530 | ➀②③④ | Single |  | Moderate |
| Sivanandy and Jie^111^ | 2021 | Malaysia | East Asia & Pacific | Cross-sectional | 98 | ② | Single | µ⮿ | Moderate |
| Riaz et al^112^ | 2020 | Pakistan | South Asia | Cross-sectional | 200 | ➀ | Single |  | High |
| Sobierajski et al^113^ | 2022 | Poland | Europe & Central Asia | Cross-sectional | 467 | ⑥ | Multiple |  | Moderate |
| Abd el aleim et al^114^ | 2023 | Egypt | Middle East & North Africa | Cross-sectional | 222 | ③ | Single |  | Moderate |
| Kahkashan et al^115^ | 2022 | India | South Asia | Cross-sectional | 90 | ➀ | Single | µ | Moderate |
| Sobierajski et al^116^ | 2021 | Poland | Europe & Central Asia | Cross-sectional | 291 | ➀ | Single |  | Moderate |
| Okedo-Alex et al^117^ | 2019 | Nigeria | Sub-Saharan Africa | Cross-sectional | 184 | ➀ | Single |  | Moderate |
| Achi et al^118^ | 2025 | Nigeria | Sub-Saharan Africa | Cross-sectional | 136 | ➀②③ | Single |  | Moderate |
| Al-Haifi et al^119^ | 2025 | Yemen | Middle East & North Africa | Cross-sectional | 600 | ➀② | Single | µ | Moderate |
| Homsi et al^120^ | 2025 | Jordan | Middle East & North Africa | Cross-sectional | 263 | ➀②④ | Single | µ | Low |
| Kumar et al^121^ | 2025 | India | South Asia | Cross-sectional | 292 | ② | Multiple | µ | Moderate |
| Saksena et al^122^ | 2025 | India | South Asia | Cross-sectional | 208 | ➀ | Single | µ | Moderate |
| Swachia et al^123^ | 2025 | India | South Asia | Cross-sectional | 337 | ➀ | Single |  | Moderate |
| Zencirci et al^124^ | 2025 | Turkey | Europe & Central Asia | Cross-sectional | 662 | ➀⑦ | Single |  | Moderate |
| Sahib et al^125^ | 2019 | Iraq | Middle East & North Africa | Cross-sectional | 146 | ② | Single | µ | High |
| Al-rudaini et al^126^ | 2025 | Oman | Middle East & North Africa | Cross-sectional | 472 | ➀ | Single |  | Moderate |
| Orok et al^127^ | 2025 | Nigeria | Sub-Saharan Africa | Pre-post study | 157 | ②⑦ | Single |  | Moderate |
| Babuya et al^128^ | 2025 | Uganda | Sub-Saharan Africa | Cross-sectional | 193 | ➀③⑦ | Single |  | Moderate |
| Ijaz et al^129^ | 2025 | Pakistan | South Asia | Cross-sectional | 379 | ➀➄⑦ | Multiple |  | Moderate |
| Narasimha Murthy et al^130^ | 2025 | India | South Asia | Cross-sectional | 500 | ➀⑦ | Single | µ | Moderate |
| Rehman et al^131^ | 2025 | Pakistan | South Asia | Cross-sectional | 1340 | ➀③➄⑦ | Multiple |  | Moderate |

Student group included: ➀Medical students, ②Pharmacy students, ③Nursing students, ④Midwifery students, ➄Dental students, ⑥Veterinary students, ⑦Others, including healthcare students whose subject areas were not defined.

Outcomes:  Cold and flu are caused by bacteria,  Antibiotics are effective in treating bacterial infections,  Antibiotics are effective against viruses,  Antibiotics are effective against cold and flu,  Treating the common cold or flu with antibiotics will speed up recovery,  Using antibiotics can lead to side effects or risks, including diarrhoea, colitis, and allergic reactions,  Patients should stop taking their prescribed/recommended antibiotics as soon as they feel better,  The unnecessary use of antibiotics makes them ineffective or leads to resistance,  Using broad-spectrum antibiotics instead of equally effective narrow-spectrum options contributes to the development of antibiotic resistance,  Resistant infections could make medical procedures like surgery, organ transplants and cancer treatment much more difficult,  Antibiotic-resistant bacteria can spread from person to person, µAwareness of antibiotic resistance, ⮿Antibiotic resistance is a public health threat worldwide.

## Supplementary Table 4: MERSQI domain and items cores for included studies.

| Domain | MERSQI item | Study  No. (%) | Maximum score | | MERSQI score  Mean (SD) | |
| --- | --- | --- | --- | --- | --- | --- |
|  |  |  | **Item** | **Domain** | **Item** | **Domain** |
| Study design | 1. Study design |  |  | 3 | 1.01 (0.08) | 1.01 (0.08) |
|  | Single-group cross-sectional or single-group post-test only | 128 (97.7) | 1 |  |  |  |
|  | Single-group pretest and post-test | 3 (2.3) | 1.5 |  |  |  |
|  | Nonrandomised, 2 group | 0 | 2 |  |  |  |
|  | Randomised controlled trial | 0 | 3 |  |  |  |
| Sampling | 1. No of institutions studied |  |  | 3 | 0.82 (0.44) | 1.76 (0.59) |
|  | 1 institution | 83 (63.4) | 0.5 |  |  |  |
|  | 2 institutions | 12 (9.1) | 1 |  |  |  |
|  | 3 or more institutions | 36 (27.5) | 1.5 |  |  |  |
|  | 1. Response rate |  |  |  | 0.94 (0.47) |  |
|  | Not applicable | 0 (0) |  |  |  |  |
|  | < 50% or not reported | 67 (51.1) | 0.5 |  |  |  |
|  | 50-74% | 11 (8.4) | 1 |  |  |  |
|  | ≥75% | 53 (40.5) | 1.5 |  |  |  |
| Type of data | 1. Type of data |  |  | 3 | 2.66 (0.75) | 2.66 (0.75) |
|  | Subjective | 22 (16.8) | 1 |  |  |  |
|  | Objective measurement | 109 (83.2) | 3 |  |  |  |
| Validity | 1. Content |  |  | 3 | 0.47 (0.50) | 0.76 (0.79) |
|  | Reported | 61 (46.6) | 1 |  |  |  |
|  | Not reported | 70 (53.4) | 0 |  |  |  |
|  | 1. Internal structure |  |  |  | 0.27 (0.44) |  |
|  | Reported | 35 (26.7) | 1 |  |  |  |
|  | Not reported | 96 (73.3) | 0 |  |  |  |
|  | 1. Relationship to other variables |  |  |  | 0.02 (0.12) |  |
|  | Reported | 2 (1.5) | 1 |  |  |  |
|  | Not reported | 129 (98.5) | 0 |  |  |  |
| Data analysis | 1. Appropriateness of data analysis |  |  | 3 | 1.00 (0) | 2.70 (0.49) |
|  | Data analysis inappropriate for study design and type of data | 0 (0) | 0 |  |  |  |
|  | Data analysis appropriate for study design and type of data | 131 (100) | 1 |  |  |  |
|  | 1. Complexity of analysis |  |  |  | 1.70 (0.49) |  |
|  | Descriptive analysis only | 38 (29.0) | 1 |  |  |  |
|  | Beyond descriptive analysis | 93 (71.0) | 2 |  |  |  |
| Outcomes | 1. Outcomes |  |  | 3 | 1.50 (0) | 1.50 (0) |
|  | Satisfaction, attitudes, perceptions, opinions, general facts | 0 | 1 |  |  |  |
|  | Knowledge, skills | 131 (100) | 1.5 |  |  |  |
|  | Behaviours | 0 | 2 |  |  |  |
|  | Patient/health care outcomes | 0 | 3 |  |  |  |

## Supplementary Table 5: Country level estimates of healthcare students with correct knowledge that antibiotics are ineffective against viruses.

| Country | Estimate | 95% Confidence Interval | |
| --- | --- | --- | --- |
|  |  | Lower limit | Upper limit |
| United Kingdom | 98.4 | 97.2 | 99.6 |
| United States | 98.0 | 94.5 | 99.3 |
| Poland | 94.0 | 90.4 | 97.5 |
| Iraq | 93.0 | 90.7 | 95.3 |
| Zambia | 92.2 | 89.8 | 94.7 |
| Jordan | 85.5 | 82.7 | 87.9 |
| Spain | 84.3 | 81.1 | 87.0 |
| Italy | 82.8 | 80.7 | 85.0 |
| West Bank and Gaza | 82.0 | 77.9 | 85.5 |
| South Africa | 80.6 | 72.8 | 86.6 |
| Mexico | 80.0 | 70.4 | 87.1 |
| Libya | 79.1 | 70.6 | 85.7 |
| Sri Lankan | 78.9 | 74.3 | 83.4 |
| Malaysia | 74.9 | 71.0 | 78.8 |
| Rwanda | 74.7 | 68.7 | 79.9 |
| Ethiopia | 70.2 | 64.0 | 75.7 |
| Japan | 70.0 | 66.4 | 73.4 |
| Pakistan | 68.7 | 49.4 | 88.1 |
| Bangladesh | 68.5 | 66.4 | 70.6 |
| India | 68.2 | 59.4 | 76.9 |
| Oman | 67.8 | 63.5 | 71.9 |
| Ghana | 66.9 | 61.0 | 72.3 |
| United Arab Emirates | 65.8 | 61.9 | 69.5 |
| Nepal | 64.7 | 55.5 | 73.9 |
| Saudi Arabia | 62.8 | 45.0 | 80.5 |
| Indonesia | 61.0 | 56.8 | 65.1 |
| Egypt | 55.4 | 48.8 | 61.8 |
| Dominica | 55.0 | 44.4 | 65.1 |
| China | 45.7 | 43.5 | 47.9 |
| Turkey | 41.0 | 38.7 | 43.3 |
| Mali | 39.9 | 35.5 | 44.5 |
| Thailand | 30.3 | 23.8 | 37.8 |

## Supplementary Table 6: Country level estimates of healthcare students with correct knowledge that antibiotics are ineffective against colds and flu.

| Country | Estimate | 95% Confidence Interval | |
| --- | --- | --- | --- |
|  |  | Lower limit | Upper limit |
| United States | 98.0 | 94.5 | 99.3 |
| Poland | 88.3 | 86.4 | 90.2 |
| Australia | 84.0 | 80.6 | 86.9 |
| United Kingdom | 81.5 | 70.9 | 92.2 |
| Serbia | 81.3 | 77.2 | 84.8 |
| Jordan | 76.7 | 73.5 | 79.7 |
| Rwanda | 74.7 | 68.7 | 79.9 |
| South Africa | 74.2 | 65.9 | 81.1 |
| Ethiopia | 70.2 | 64.0 | 75.7 |
| India | 66.1 | 52.8 | 79.3 |
| Malaysia | 65.6 | 58.9 | 71.8 |
| Trinidad and Tobago | 62.0 | 52.6 | 70.6 |
| South Korea | 62.0 | 56.3 | 67.4 |
| Sudan | 61.5 | 58.8 | 64.2 |
| Sri Lankan | 60.1 | 45.7 | 74.5 |
| Spain | 59.2 | 55.2 | 63.1 |
| Japan | 55.8 | 52.7 | 58.9 |
| Dominica | 55.0 | 44.4 | 65.1 |
| Oman | 54.7 | 50.2 | 59.1 |
| Pakistan | 49.1 | 32.7 | 65.5 |
| Italy | 43.0 | 34.0 | 52.5 |
| Ghana | 40.0 | 37.1 | 43.0 |
| Saudi Arabia | 38.6 | 34.9 | 42.4 |
| Nigeria | 33.9 | 31.1 | 36.8 |
| Mali | 32.1 | 27.9 | 36.6 |
| Thailand | 29.7 | 27.2 | 32.4 |
| Egypt | 27.5 | 22.1 | 33.7 |
| Iraq | 21.8 | 15.6 | 29.6 |

## Supplementary Table 7: Country level estimates of healthcare students with correct knowledge that antibiotics that antibiotics do not speed up the recovery from the common cold and flu

| Country | Estimate | 95% Confidence Interval | |
| --- | --- | --- | --- |
|  |  | Lower limit | Upper limit |
| United States | 98.0 | 94.5 | 99.3 |
| United Kingdom | 97.0 | 93.4 | 98.7 |
| Spain | 94.5 | 92.3 | 96.1 |
| Iraq | 84.3 | 80.5 | 88.1 |
| Uganda | 77.4 | 74.7 | 80.2 |
| Malaysia | 75.4 | 69.8 | 80.2 |
| Zambia | 65.0 | 60.8 | 69.3 |
| Bangladesh | 56.8 | 54.2 | 59.4 |
| India | 42.7 | 29.4 | 56.1 |
| Nepal | 41.2 | 34.0 | 48.3 |
| China | 37.5 | 35.9 | 39.0 |
| Pakistan | 37.3 | 33.9 | 40.8 |
| Ethiopia | 35.0 | 30.0 | 40.4 |
| United Arab Emirates | 34.2 | 30.5 | 38.1 |
| Saudi Arabia | 33.5 | 28.3 | 39.2 |
| Turkey | 28.8 | 26.6 | 31.0 |
| Sri Lankan | 17.7 | 10.8 | 27.6 |

## Supplementary Table 8: Heterogeneity and Egger’s bias estimates across studies

| Knowledge area | N | Pooled estimate | Heterogeneity | Egger’s bias |
| --- | --- | --- | --- | --- |
|  |  | Correct Knowledge  % (95% CI) | *I^2^* (*P*-value) | Estimate (95%CI, P-value) |
| Antibiotics are effective in treating bacterial infections | 57 | 89.1 (87.4 – 90.9) | 97.7% (< 0.001) | -7.3 (-20.0 to -4.5, P < 0.001) |
| Antibiotics are effective against viruses. | 73 | 70.3 (65.9 – 74.7) | 99.2% (< 0.001) | -9.1 (-13.9 to -4.4, P < 0.001) |
| Antibiotics are effective against cold and flu. | 49 | 58.7 (52.2 – 65.2) | 99.3% (< 0.001) | -10.4 (-17.0 to -3.9, P = 0.002) |
| Treating the common cold or flu with antibiotics will speed up recovery | 34 | 51.5 (41.6 – 61.4) | 99.6% (< 0.001) | -4.9 (-15.8 to 5.9, P = 0.362) |
| Bacteria cause the common cold and flu | 11 | 57.4 (49.0–65.8) | 99.6% (< 0.001) | -5.0 (-13.9 to 3.9, P = 0.227) |
| Using antibiotics can lead to side effects or risks, including diarrhoea, colitis, and allergic reactions | 42 | 78.8 (74.6 – 83.1) | 98.7% (< 0.001) | -9.2 ( -14.5 to -3.9. P = 0.001) |
| Using broad-spectrum antibiotics instead of equally effective narrow-spectrum options contributes to the development of antibiotic resistance. | 19 | 71.3 (65.9 – 76.8) | 96.3% (< 0.001) | 0.94 (-6.6 to 8.5, P = 0.795) |
| The unnecessary use of antibiotics makes them ineffective or leads to resistance | 63 | 87.0 (85.1 – 89.0) | 98.1% (< 0.001) | 7.6 (-9.5 to -5.7, P < 0.001) |
| Patients should stop taking their prescribed antibiotics as soon as they feel better | 42 | 76.2 (72.2 – 80.2) | 98.0% (< 0.001) | -10.0 (-12.9 to -7.0, P < 0.001) |
| Resistant infections could make medical procedures like surgery, organ transplants and cancer treatment much more difficult | 9 | 76.1 (67.0 – 85.3) | 98.3% (< 0.001) | -10.6 (-21.1 to -0.1, P = 0.048) |
| Antibiotic resistant bacteria can spread from person to person. | 10 | 62.2 (52.4 – 72.0) | 98.1% (< 0.001) | -5.9 (-21.1 to 9.3, P = 0.399) |

## References

1. Abdelkarim OA, Abubakar U, Hussain MA, Abadi AEB, Mohamed AO, Osman W, et al. Knowledge, Perception, and Self-Confidence of Antibiotic Resistance, Appropriate Antibiotic Therapy, and Antibiotic Stewardship Among Undergraduate Pharmacy Students in Sudan. Infection and Drug Resistance. 2024;17:935-49.

2. Abdu-Aguye SN, Barde KG, Yusuf H, Lawal BK, Shehu A, Mohammed E. Investigating Knowledge of Antibiotics, Antimicrobial Resistance and Antimicrobial Stewardship Concepts Among Final Year Undergraduate Pharmacy Students in Northern Nigeria. Integrated Pharmacy Research and Practice. 2022;11:187-95.

3. Abuawad M, Ziyadeh-Isleem A, Mahamid A, Quzmar S, Ammar E, Shawahna R. Knowledge, perception, and attitudes of medical students towards antimicrobial resistance and stewardship: an observational cross-sectional study from Palestine. BMC Med Educ. 2024;24(1):302.

4. Abubakar U, Muhammad HT, Sulaiman SAS, Ramatillah DL, Amir O. Knowledge and self-confidence of antibiotic resistance, appropriate antibiotic therapy, and antibiotic stewardship among pharmacy undergraduate students in three Asian countries. Curr Pharm Teach Learn. 2020;12(3):265-73.

5. Ahmad A, Khan MU, Patel I, Maharaj S, Pandey S, Dhingra S. Knowledge, attitude and practice of B.Sc. Pharmacy students about antibiotics in Trinidad and Tobago. J Res Pharm Pract. 2015;4(1):37-41.

6. Ahmad A, Khan MU, Moorthy J, Jamshed SQ, Patel I. Comparison of knowledge and attitudes about antibiotics and resistance, and antibiotics self-practicing between Bachelor of Pharmacy and Doctor of Pharmacy students in Southern India. Pharm Pract (Granada). 2015;13(1):523.

7. Akande-Sholabi W, Ajamu AT. Antimicrobial stewardship: Assessment of knowledge, awareness of antimicrobial resistance and appropriate antibiotic use among healthcare students in a Nigerian University. BMC medical education. 2021;21(1):488.

8. Akbar Z, Alquwez N, Alsolais A, Thazha SK, Ahmad MD, Cruz JP. Knowledge about antibiotics and antibiotic resistance among health-related students in a Saudi University. Journal of Infection in Developing Countries. 2021;15(7):925-33.

9. Al-Qerem W, Hammad A, Jarab A, M MS, Amawi HA, Ling J, et al. Knowledge, attitudes, and practice with respect to antibiotic use among pharmacy students: a cross-sectional study. Eur Rev Med Pharmacol Sci. 2022;26(10):3408-18.

10. Al-Sadoon MA. Knowledge of medical students regards antibiotic consumption for upper respiratory tract infections in Basra. Annals of Tropical Medicine and Public Health. 2020;23(13).

11. Alsaleh N, Alsmari A, Alanazi F, Alsaleh A, Alsmari R, Al-Swedan N. Medical and dental students' knowledge and perceptions about antimicrobial stewardship: a call for educational enhancement. Military Medical Science Letters (Vojenske Zdravotnicke Listy). 2020;89(4):207-14.

12. Alsayed AR, Darwish El Hajji F, Al-Najjar MAA, Abazid H, Al-Dulaimi A. Patterns of antibiotic use, knowledge, and perceptions among different population categories: A comprehensive study based in Arabic countries. Saudi Pharmaceutical Journal. 2022;30(3):317-28.

13. Al-Taani GM, Karasneh RA, Al-Azzam S, Bin Shaman M, Jirjees F, Al-Obaidi H, et al. Knowledge, Attitude, and Behavior about Antimicrobial Use and Resistance among Medical, Nursing and Pharmacy Students in Jordan: A Cross Sectional Study. Antibiotics. 2022;11(11).

14. Assar A, Abdelraoof MI, Abdel-Maboud M, Shaker KH, Menshawy A, Swelam AH, et al. Knowledge, attitudes, and practices of Egypt's future physicians towards antimicrobial resistance (KAP-AMR study): a multicenter cross-sectional study. Environmental science and pollution research international. 2020;27(17):21292-8.

15. Augie BM, Van Zyl RL, McInerney PA, Miot J. Knowledge and perceptions about antibiotic resistance and prudent antibiotic prescribing among final year medical students in two African countries. International Journal of Pharmacy Practice. 2021;29(5):508-14.

16. Azechi T, Sasano H, Sato K, Arakawa R, Suzuki K. Evaluation of Knowledge Regarding the Use of Antibiotics among Pharmacy Undergraduates in Japan. J Microbiol Biol Educ. 2022;23(3).

17. Bharti RK, Pathania JS, Sood V, Koshewara P, Dewangan T. Assessing the Knowledge, Attitude, and Practice (KAP) of Antimicrobial Resistant among MBBS, BDS and BSc Nursing Students in the Northern State of India. An Observational-based Cross-sectional Study. Advances in Bioscience and Clinical Medicine. 2020;8(2):10-6.

18. Bonna AS, Mazumder S, Manna RM, Pavel SR, Nahin S, Ahmad I, et al. Knowledge attitude and practice of antibiotic use among medical students in Bangladesh: A cross-sectional study. Health Science Reports. 2024;7(9).

19. Bukhsh A, Hussain S, Rehman IU, Mallhi TH, Khan YH, Khaliel AM, et al. Awareness and perception of seasonal influenza (Flu) among health science and Non-Health science university students in Pakistan: A nationwide survey. Pak J Pharm Sci. 2019;32(4(Supplementary)):1789-96.

20. Chakravarty A, Nabi N, Basu R, Gupta P, Raja D, Dutta R, et al. A Multicentric Survey of Indian Medical Students about their Knowledge and Perception on Antimicrobial Stewardship. Journal of Pure and Applied Microbiology. 2022;16(2):1049-59.

21. Choi JS, Kim KY. Factors associated with preventive behaviors in the overuse and misuse of antibiotics in Korean nursing students. Am J Infect Control. 2019;47(6):715-7.

22. Elmahi OKO, Musa RAE, Shareef AAH, Omer MEA, Elmahi MAA, Altamih RAA, et al. Perception and practice of self-medication with antibiotics among medical students in Sudanese universities: A cross-sectional study. PLoS ONE. 2022;17(1 January).

23. Fetensa G, Wakuma B, Tolossa T, Fekadu G, Bekuma TT, Fayisa L, et al. Knowledge and Attitude Towards Antimicrobial Resistance of Graduating Health Science Students of Wollega University. Infect Drug Resist. 2020;13:3937-44.

24. Gayathri PS, Krithika C, Sandhya V, Sangeetha E, Santhoshika T, Ramani P. Knowledge and attitude towards antibiotic prescription among dental students. Indian Journal of Forensic Medicine and Toxicology. 2021;15(2):462-6.

25. Hagiya H, Ino H, Tokumasu K, Ogawa H, Miyoshi T, Ochi K, et al. Antibiotic literacy among Japanese medical students. Journal of Infection and Chemotherapy. 2020;26(10):1107-9.

26. Haque M, Rahman NAA, McKimm J, Sartelli M, Kibria GM, Islam MZ, et al. Antibiotic Use: A Cross-Sectional Study Evaluating the Understanding, Usage and Perspectives of Medical Students and Pathfinders of a Public Defence University in Malaysia. Antibiotics (Basel). 2019;8(3).

27. Harakeh S, Almatrafi M, Ungapen H, Hammad R, Olayan F, Hakim R, et al. Perceptions of medical students towards antibiotic prescribing for upper respiratory tract infections in Saudi Arabia. BMJ Open Respir Res. 2015;2(1):e000078.

28. Hayat K, Jamshed S, Rosenthal M, Haq NU, Chang J, Rasool MF, et al. Understanding of pharmacy students towards antibiotic use, antibiotic resistance and antibiotic stewardship programs: A cross-sectional study from punjab, pakistan. Antibiotics. 2021;10(1):1-13.

29. Hayat K, Fatima N, Umer MF, Khan FU, Khan FU, Najeeb ZuR, et al. Understanding of future prescribers about antimicrobial resistance and their preparedness towards antimicrobial stewardship activities in Pakistan: Findings and implications. Frontiers in Pharmacology. 2022;13:771083.

30. Higuita-Gutiérrez LF, Roncancio Villamil GE, Jiménez Quiceno JN. Knowledge, attitude, and practice regarding antibiotic use and resistance among medical students in Colombia: a cross-sectional descriptive study. BMC Public Health. 2020;20(1):1861.

31. Hu Y, Wang X, Tucker JD, Little P, Moore M, Fukuda K, et al. Knowledge, attitude, and practice with respect to antibiotic use among Chinese medical students: A multicentre cross-sectional study. International Journal of Environmental Research and Public Health. 2018;15(6).

32. Hussain I, Yousaf N, Haider S, Jalil P, Saleem MU, Imran I, et al. Assessing Knowledge and Perception Regarding Antimicrobial Stewardship and Antimicrobial Resistance in University Students of Pakistan: Findings and Implications. Antibiotics (Basel). 2021;10(7).

33. Hussain M, Atif MA, Akhtar L. Impact of antibiotic stewardship learning session on knowledge and attitude of undergraduate medical students. Khyber Medical University Journal. 2021;13(3):152-6.

34. Inácio J, Barnes LM, Jeffs S, Castanheira P, Wiseman M, Inácio S, et al. Master of Pharmacy students’ knowledge and awareness of antibiotic use, resistance and stewardship. Currents in Pharmacy Teaching and Learning. 2017;9(4):551-9.

35. Jairoun A, Hassan N, Ali A, Jairoun O, Shahwan M. Knowledge, attitude and practice of antibiotic use among university students: a cross sectional study in UAE. BMC Public Health. 2019;19(1):518.

36. Jayawardhana Y, Premaratne A, Kalpani S, Jayasundara S, Jayawardhane G, Jayawarna C, et al. Investigating the drivers for antibiotic use and misuse amongst medical undergraduates-perspectives from a Sri Lankan medical school. PLOS Glob Public Health. 2023;3(3):e0001740.

37. Jayaweerasingham M, Angulmaduwa S, Liyanapathirana V. Knowledge, beliefs and practices on antibiotic use and resistance among a group of trainee nurses in Sri Lanka. BMC research notes. 2019;12(1):601.

38. Jianvitayakij S, Niyomyart A, Junsawang C, Bualoy W, Butsing N, Monkong S, et al. Knowledge of antibiotics and antibiotic resistance, antibiotic use and eHealth literacy among nursing students in Thailand: a cross-sectional study. BMJ Open. 2024;14(11):e090956.

39. Kandasamy G, Sivanandy P, Almaghaslah D, Khobrani M, Chinnadhurai M, Vasudevan R, et al. Knowledge, attitude, perception and practice of antibiotics usage among the pharmacy students. International Journal of Clinical Practice. 2020;74(11).

40. Kanyike AM, Olum R, Kajjimu J, Owembabazi S, Ojilong D, Nassozi DR, et al. Antimicrobial resistance and rational use of medicine: knowledge, perceptions, and training of clinical health professions students in Uganda. Antimicrobial Resistance and Infection Control. 2022;11(1).

41. Karthik S, Kavya S, Kanchana M, Karthick N, Krithika S, Ragesh G, et al. A study on knowledge, attitude and practice of antibiotic usage among pharmacy students in college of pharmacy, Sri Ramachandra Medical College and Research Institute (DU): An descriptive analysis. International Research Journal of Pharmacy. 2019;10(5):98-102.

42. Sharon Keziah V, Pandurangan KK. A cross sectional survey of knowledge, attitude and practice of antibiotic use among dental and paramedical students. International Journal of Pharmaceutical Research. 2020;12:3489-506.

43. Khajuria K, Kaur S, Sadiq S, Khajuria V. KAP on antibiotic usage and resistance among second professional medical students. International Journal of Basic & Clinical Pharmacology. 2019;8(1):68-74.

44. Khan FU, Khan A, Shah S, Hayat K, Usman A, Khan FU, et al. Exploring Undergraduate Pharmacy Students Perspectives Towards Antibiotics Use, Antibiotic Resistance, and Antibiotic Stewardship Programs Along With the Pharmacy Teachers’ Perspectives: A Mixed-Methods Study From Pakistan. Frontiers in Pharmacology. 2021;12.

45. Khanikar D, Singh SR, Bhattacharyya M, Saikia PP, Patowary SS. TO ASSESS THE KNOWLEDGE, ATTITUDE AND PRACTICE OF ANTIBIOTIC USAGE AND RESISTANCE AMONG THE UNDERGRADUATE MEDICAL STUDENTS IN A TERTIARY CARE TEACHING HOSPITAL IN ASSAM: A QUESTIONNAIRE-BASED STUDY. International Journal of Pharmacy and Pharmaceutical Sciences. 2024;16(10):21-5.

46. Lubwama M, Onyuka J, Ayazika KT, Ssetaba LJ, Siboko J, Daniel O, et al. Knowledge, attitudes, and perceptions about antibiotic use and antimicrobial resistance among final year undergraduate medical and pharmacy students at three universities in East Africa. PLoS ONE. 2021;16(5 May).

47. Manning ML, Jack D, Wheeler L, Okupniak C, Pogorzelska-Maziarz M. Effect of a virtual simulated participant experience on antibiotic stewardship knowledge among pre-licensure baccalaureate nursing students: A pilot study. Nurse Educ Today. 2022;113:105362.

48. Mech K, Borah M, Das BR. KNOWLEDGE OF ANTIMICROBIAL RESISTANCE AMONG UNDERGRADUATE MBBS STUDENTS IN A TERTIARY CARE TEACHING HOSPITAL, ASSAM. International Journal of Academic Medicine and Pharmacy. 2023;5(5):1078-83.

49. Meher B, Srinivasan A, Vighnesh C, Padhy B, Mohanty R. Factors most influencing antibiotic stewardship program and comparison of prefinal- and final-year undergraduate medical students. Perspectives in Clinical Research. 2020;11(1):18-23.

50. Mohan Kumar KS, Kishore MS, Hemanth Kumar KH. Knowledge, attitude, and practice toward antibiotic use and antibiotic resistance among 2nd-year medical students: A cross-sectional study. National Journal of Physiology, Pharmacy and Pharmacology. 2024;14(1):42-6.

51. Mubarak A, Alwafi MM, Alharbi RM, Alserhani SA, Khushaim RF, Almadani GZ, et al. Knowledge and Attitude Toward Antibiotic Prescription Among Dental Students and Interns at Multiple Universities in Saudi Arabia. Cureus. 2024;16(1):e51777.

52. Nisabwe L, Brice H, Umuhire MC, Gwira O, Harelimana JD, Nzeyimana Z, et al. Knowledge and attitudes towards antibiotic use and resistance among undergraduate healthcare students at University of Rwanda. J Pharm Policy Pract. 2020;13:7.

53. Nowbuth AA, Monteiro FJ, Sheets LR, Asombang AW. Assessment of the knowledge, attitudes and perceived quality of education about antimicrobial use and resistance of medical students in Zambia, Southern Africa. JAC-Antimicrobial Resistance. 2023;5(3).

54. Nukaly HY, Aljuhani RA, Alhartani MM, Alhindi YZ, Asif U, Alshanberi AM, et al. Knowledge of Antibiotic Use and Resistance Among Medical Students in Saudi Arabia. Adv Med Educ Pract. 2024;15:501-12.

55. Owusu-Ofori AK, Darko E, Danquah CA, Agyarko-Poku T, Buabeng KO. Self-Medication and Antimicrobial Resistance: A Survey of Students Studying Healthcare Programmes at a Tertiary Institution in Ghana. Frontiers in public health. 2021;9:706290.

56. Pradhan A, Mishra RS, Tamang B, Sharma K, Dewan S, Sharma B, et al. Assessment of knowledge, attitude, and practice of pharmacy students on antibiotic use and its resistance: A cross-sectional study. International Journal of Green Pharmacy. 2024;18(3):196-202.

57. Precha N, Sukmai S, Hengbaru M, Chekoh M, Laohaprapanon S, Makkaew P, et al. Knowledge, attitudes, and practices regarding antibiotic use and resistance among health science and non-health science university students in Thailand. PLoS ONE. 2024;19(1 January).

58. Rábano-Blanco A, Domínguez-Martís EM, Mosteiro-Miguéns DG, Freire-Garabal M, Novío S. Nursing students’ knowledge and awareness of antibiotic use, resistance and stewardship: A descriptive cross-sectional study. Antibiotics. 2019;8(4).

59. Raees I, Atif HM, Aslam S, Mustafa ZU, Meyer JC, Hayat K, et al. Understanding of Final Year Medical, Pharmacy and Nursing Students in Pakistan towards Antibiotic Use, Antimicrobial Resistance and Stewardship: Findings and Implications. Antibiotics. 2023;12(1).

60. Rajiah K, Ren WS, Jamshed SQ. Evaluation of the understanding of antibiotic resistance among Malaysian pharmacy students at public universities: An exploratory study. Journal of Infection and Public Health. 2015;8(3):266-73.

61. Reena AP, Ittyachen AM. Awareness of Antibiotic Resistance among Medical Students in Kerala State, India: A Cross‑Sectional Study. Current Medical Issues. 2022;20(4):245-52.

62. Saikia AL, Saikia H, Borah A, Chaliha M. Assessment of knowledge, attitude, and practice of rational antibiotic use among medical students in a tertiary care teaching hospital of upper Assam – A cross-sectional study. National Journal of Physiology, Pharmacy and Pharmacology. 2024;14(3):580-6.

63. Sakeena MH, Bennett AA, Mohamed F, Herath HM, Gawarammane I, McLachlan AJ. Investigating knowledge regarding antibiotics among pharmacy and allied health sciences students in a Sri Lankan university. J Infect Dev Ctries. 2018;12(9):726-32.

64. Sakeena MHF, Bennett AA, McLachlan AJ. Investigating knowledge regarding antibiotics and antimicrobial resistance among pharmacy students in Australian universities. Journal of Pharmacy Practice and Research. 2021;51(1):54-61.

65. Sannathimmappa MB, Nambiar V, Aravindakshan R. A cross-sectional study to evaluate the knowledge and attitude of medical students concerning antibiotic usage and antimicrobial resistance. International Journal of Academic Medicine. 2021;7(2):113-9.

66. Scaioli G, Gualano MR, Gili R, Masucci S, Bert F, Siliquini R. Antibiotic Use: A cross-sectional survey assessing the knowledge, attitudes and practices amongst students of a school of medicine in Italy. PLoS ONE. 2015;10(4).

67. Seid MA, Hussen MS. Knowledge and attitude towards antimicrobial resistance among final year undergraduate paramedical students at University of Gondar, Ethiopia. BMC Infectious Diseases. 2018;18(1).

68. Shah P, Shrestha R, Mao Z, Chen Y, Chen Y, Koju P, et al. Knowledge, Attitude, and Practice Associated with Antibiotic Use among University Students: A Survey in Nepal. Int J Environ Res Public Health. 2019;16(20).

69. Shahnaz S, Khan MA, Zeb Un n, Alam S, Ali SI, Khatian N, et al. Knowledge Attitude and Practice (KAP) of Pharmacy students regarding the use of Antibiotics in Karachi. Archives of Pharmacy Practice. 2020;11(4):135-40.

70. Sharma AK, Goyal V, Sankhla S. KNOWLEDGE, ATTITUDE AND PRACTICE TOWARDS ANTIBIOTIC USE AND ANTIBIOTIC RESISTANCE AMONG MEDICAL STUDENTS: A CROSS-SECTIONAL STUDY. International Journal of Pharmaceutical Sciences and Research. 2022;13(10):4166-72.

71. Shrestha R. Knowledge, attitude and practice on antibiotics use and its resistance among medical students in a tertiary care hospital. Journal of the Nepal Medical Association. 2019;57(216):74-9.

72. Sowmya CN, Surekha A, Latha NS, Mohiddin SK. A STUDY ON KNOWLEDGE, ATTITUDE, AND PRACTICE REGARDING ANTIBIOTIC USE AND RESISTANCE AMONG MEDICAL STUDENTS. Journal of Population Therapeutics and Clinical Pharmacology. 2024;31(5):198-202.

73. Struzycka I, Mazinska B, Bachanek T, Boltacz-Rzepkowska E, Drozdzik A, Kaczmarek U, et al. Knowledge of antibiotics and antimicrobial resistance amongst final year dental students of Polish medical schools-A cross-sectional study. Eur J Dent Educ. 2019;23(3):295-303.

74. Tariq K, Hassan M, Wajahat M, Muneer N, Imran E. Awareness of antibiotic use and antibiotic resistance amongst dental students. Brazilian Dental Science. 2021;24(3).

75. Tayyab K, Shahzadi I, Mukhtar F, Ali Shahid H, Tahir I, Gohar H. Knowledge and perceptions of medical students regarding antibiotic use. Pakistan Journal of Medical and Health Sciences. 2017;11(1):145-9.

76. Teague E, Bezuidenhout S, Meyer JC, Godman B, Engler D. Knowledge and Perceptions of Final-Year Nursing Students Regarding Antimicrobials, Antimicrobial Resistance, and Antimicrobial Stewardship in South Africa: Findings and Implications to Reduce Resistance. Antibiotics. 2023;12(12).

77. Yadav H, Kaur S, Kawle S, Jhalani G. A Study of Knowledge and Attitude and Practice of Antimicrobials and Antimicrobial Resistance among Medical Students. International Journal of Pharmaceutical and Clinical Research. 2024;16(6):2166-72.

78. Yahya RN, Wasmi MM. A comparative study about knowledge, attitude, practice of antibiotic use and perceptions of the possible causes of resistance between final year undergraduate students and postgraduate pharmacy students. Medico-Legal Update. 2021;21(2):1030-7.

79. Yang C, Xie J, Chen Q, Yuan Q, Shang J, Wu H, et al. Knowledge, Attitude, and Practice About Antibiotic Use and Antimicrobial Resistance Among Nursing Students in China: A Cross Sectional Study. Infection and Drug Resistance. 2024;17:1085-98.

80. Zawahir S, Hettiarachchi C, Morrissey H. Assessing knowledge, perception and attitudes about antibiotics among final year pharmacy undergraduates in Sri Lanka. International Journal of Pharmacy and Pharmaceutical Sciences. 2017;9(11):234-9.

81. Chen J, Sidibi AM, Shen X, Dao K, Maiga A, Xie Y, et al. Lack of antibiotic knowledge and misuse of antibiotics by medical students in Mali: a cross-sectional study. Expert Review of Anti-Infective Therapy. 2021;19(6):797-804.

82. Golding SE, Higgins HM, Ogden J. Assessing Knowledge, Beliefs, and Behaviors around Antibiotic Usage and Antibiotic Resistance among UK Veterinary Students: A Multi-Site, Cross-Sectional Survey. Antibiotics. 2022;11(2).

83. Betancourt SDP, Peña SDP, Parra-Forero LY. The knowledge of antibiotics in veternary students and repercution in human health. Health. 2020;12(12):1632.

84. Chapot L, Sarker MS, Begum R, Hossain D, Akter R, Hasan MM, et al. Knowledge, attitudes and practices regarding antibiotic use and resistance among veterinary students in Bangladesh. Antibiotics. 2021;10(3) (no pagination).

85. Almadah R, Alazraq H, Khetresh M, Gazeti S. Knowledge, Attitude and Practices Regarding Antibiotics usages among Medical Students at Zawia University. AlQalam Journal of Medical and Applied Sciences. 2024:916-26.

86. Corrente M, Trotta A, Marinaro M, Cavalli A, Lovreglio P, Cirilli M, et al. Basic knowledge and misconceptions on antibiotic use: a comparative survey between Veterinary College and High School students in Bari (Italy). Veterinaria Italiana. 2021;57(2):127-34.

87. Dass E, Patel A, Patel H, Patel D, Patel H, Patel H, et al. A cross-sectional questionnaire-based study of knowledge, attitude and practice of antibiotic usage among the undergraduate students of a tertiary care teaching rural hospital: with an emphasis of WHO fact-sheets. International Journal of Basic & Clinical Pharmacology. 2019;8(9):2113.

88. De M, Mukherjee D. Antibiotic Use: Knowledge and Practice of Medical Undergraduate Students in Kolkata. Bengal Journal of Otolaryngology and Head Neck Surgery. 2020;28(1):1-9.

89. Doe P, Danquah CA, Ohemeng KA, Mashood GA, Sepenoo J, Buabeng KO, et al. Evaluation of the Knowledge, Attitude and Perception of Healthcare Students on Antibiotics and Antibiotic Resistance: A Study in Central University, Ghana. Journal of Tropical Pharmacy and Chemistry. 2023;7(2):59-66.

90. Dyar OJ, Hills H, Seitz LT, Perry A, Ashiru-Oredope D. Assessing the knowledge, attitudes and behaviors of human and animal health students towards antibiotic use and resistance: a pilot cross-sectional study in the UK. Antibiotics. 2018;7(1).

91. Emera NM, El-Baraky IA, Abbassi MM, Sabry NA. Knowledge, Attitude, and Practice Towards Antibiotics Use Among Medical Sector Final-Year Students in Egypt. Med Sci Educ. 2024;34(6):1369-79.

92. Gupta RK, Singh P, Rani R, Kumari R, Langer B, Gupta R. Antibiotic use: evaluating knowledge, attitude and practices among medical students in a sub Himalayan state. Int J Basic Clin Pharmacol. 2017;6(10):2516-21.

93. Hameed ALAS. Antibiotic knowledge and practices of students attending in Medical Institute/Southern Technical University. Al-Salam Journal for Medical Science. 2024;3(1):68-74.

94. Horvat O, Petrović AT, Paut Kusturica M, Bukumirić D, Jovančević B, Kovačević Z. Survey of the Knowledge, Attitudes and Practice towards Antibiotic Use among Prospective Antibiotic Prescribers in Serbia. Antibiotics. 2022;11(8).

95. Kanneppady SS, Oo AM, Lwin OM, Al-Abed A-AAA, Kanneppady SK. Knowledge, attitude, and awareness of antibiotic resistance among medical students. Archives of Medicine and Health Sciences. 2019;7(1):57-60.

96. Lomi M, Jimsha V, Srinivasan S, Daniel MJ, Territory U. Assessment of knowledge, attitude and practice of antibiotic usage amongst undergraduate, intern and postgraduate dental students-a questionnaire based study. Int J Sci Healthcare Res. 2019;4:136-42.

97. Magbol M, Osman M, Ahmed M, Alneama M, Hassieb M. Assessment of Knowledge, Attitude and Practice of Antibiotic Resistance among Medical Student at Al-Zaiem Al-Azhari University, December 2021–July 2022. Int Internal Med J. 2024;2(1):01-6.

98. Marta-Costa A, Miranda C, Silva V, Silva A, Martins Â, Pereira JE, et al. Survey of the knowledge and use of antibiotics among medical and veterinary health professionals and students in portugal. International Journal of Environmental Research and Public Health. 2021;18(5):1-14.

99. Memon I, Alrashid AA, Alshammari HS, Rehman DE, Feroz Z, Nagro A, et al. Assessing the Effects of Basic Medical Science Courses on the Knowledge and Attitude towards Antibiotic Usage among Pre-Professional Students in Saudi Arabia. Pharmacy (Basel). 2021;9(2).

100. Mudenda S, Mukela M, Matafwali S, Banda M, Mutati RK, Muungo LT, et al. Knowledge, attitudes, and practices towards antibiotic use and antimicrobial resistance among pharmacy students at the University of Zambia: implications for antimicrobial stewardship programmes. Scholars Academic Journal of Pharmacy. 2022;11(8):117-24.

101. Nair CC, Mahadeven S, Asheeta A, Hima C, Beena M, Ajitha J. Knowledge, attitude and practices related to antibiotic use among pharmacy students in South India. World Journal of Pharmaceutical Research. 2020;9:13.

102. Nayak S, Rana M, Mayya SS, Gupta GP, Wazir SS, Parmar KS, et al. Antibiotics to cure or harm: Concept of antibiotic resistance among health professional students in Nepal. International Journal of Medical Science and Public Health. 2016;5(12):2512-7.

103. Nwafia I, Nwafia S, Ibeh P, Ajunwa K, Obaje O, Ehimiyen R, et al. Knowledge of antibiotic resistance and habits of antibiotic use among medical students of University of Nigeria Enugu: a descriptive cross-sectional survey: Connaissance de la résistance aux antibiotiques et des habitudes d'utilisation des antibiotiques parmi les étudiants en médecine de l'Université du Nigéria à Enugu: une enquête transversale descriptive. African Journal of Clinical and Experimental Microbiology. 2024;25(3):342-9.

104. Olatunde SK, Owolabi JB. Knowledge, attitudes and practices of antibiotic use and antimicrobial resistance among medical students in a private university in Dominica, West Indies. Advances in Microbiology. 2022;12(9):511-24.

105. Orhan Z. An Evaluation of the Knowledge, Attitude, and Behaviour to Pharmacovigilance in the Use of Antibiotics of Students in the Health Sciences Field. International Journal of Nursing Student Scholarship. 2022;9:1-14.

106. Panthi S, Pathak P, Sitaula J. Knowledge, attitude and practice on antibiotic use and its resistance among medical students in a tertiary care hospital. Journal of Chitwan Medical College. 2020;10(4):16-9.

107. Sakr S, Ghaddar A, Hamam B, Sheet I. Antibiotic use and resistance: an unprecedented assessment of university students' knowledge, attitude and practices (KAP) in Lebanon. BMC Public Health. 2020;20(1):535.

108. Zulu A, Matafwali SK, Banda M, Mudenda S. Assessment of knowledge, attitude and practices on antibiotic resistance among undergraduate medical students in the school of medicine at the University of Zambia. Int J Basic Clin Pharmacol. 2020;9(2):263-70.

109. Kaushik T, Setya S. Assessment of Knowledge, Attitude and Practice Towards Antibiotics among Medical and Dental Students in Indian University: A Cross-Sectional Study. J Clin Biomed Sci. 2024;14(4):148-58.

110. Syahniar R, Farsida ANNK, Mardhia M, Bekti HS, Marpaung NLE, Dharmawan A, et al. Knowledge and attitudes towards antibiotic resistance among health profession students in Indonesia. International Journal of Public Health. 2024;13(1):404-12.

111. Sivanandy P, Jie TS. Knowledge, Attitude, Perception and Practice of Pharmacy Students Concerning Antibiotic Use and Antibiotic Resistance. J Clin Pharmacol Ther. 2021;2(2):1015.

112. Riaz MMA, Shahid R, Naeem A, Ehsan SB, Ali S, Saleem M. Knowledge, Attitude and Behavior regarding Antibiotics Misuse and Use among Medical Students of Public Sector Medical College of Faisalabad. Annals of Punjab Medical College. 2020;14(2):164-7.

113. Sobierajski T, Mazińska B, Chajęcka-Wierzchowska W, Śmiałek M, Hryniewicz W. Antimicrobial and Antibiotic Resistance from the Perspective of Polish Veterinary Students: An Inter-University Study. Antibiotics. 2022;11(1).

114. Abd El Aleim R, Mohamed AE-H, Mohamed Al Sherbeny E. Knowledge and Practices of Nursing Students regarding Risks of Antibiotics Self-Medication in Beni-Suef City. Egyptian Journal of Health Care. 2023;14(3):1169-91.

115. Kahkashan I, Farooq M, Imran S. Assessment of Knowledge, Attitude and Practices about Antibiotic Resistance and Usage: A Questionnaire Based Study Among Medical Students. International journal of health sciences.6(S1):608-16.

116. Sobierajski T, Mazińska B, Wanke-Rytt M, Hryniewicz W. Knowledge-based attitudes of medical students in antibiotic therapy and antibiotic resistance. A cross-sectional study. International Journal of Environmental Research and Public Health. 2021;18(8).

117. Okedo-Alex I, Madubueze UC, Umeokonkwo CD, Oka OU, Adeke AS, Okeke KC. Knowledge of antibiotic use and resistance among students of a medical school in Nigeria. Malawi medical journal : the journal of Medical Association of Malawi. 2019;31(2):133-7.

118. Achi CJ, David TO, Ovwasa FT, Ejenavwo E, Gbagbeke KO, Owolabi SO. The knowledge and perception of undergraduate students on antimicrobial resistance and antimicrobial stewardship in selected faculties in Delta state university, Abraka. World Journal of Biology Pharmacy and Health Sciences. 2025;21(1):398-407.

119. Al-Haifi AY, Al-Shami AS, Al-Akhali KM, Al-Mehdar AA. Knowledge, Attitude and Practice of Antimicrobial Usage Among Undergraduate Medical Students in Universities and Institutes, Thamar, Yemen. Infect Drug Resist. 2025;18:1675-86.

120. Homsi SR, Alkhaldi SM, Taha HA, Homsi ZR. Healthcare Students' Knowledge, Attitudes, and Practices Related to Antibiotic Resistance and Use in Jordan: A Cross-Sectional Study. J Med Educ Curric Dev. 2025;12:23821205251344732.

121. Kumar BP, Battula P, Basha MS, Murthi KEK, Narasimha VL, Prasad RD, et al. A questionnaire-based study to assess the knowledge, attitude, and practices of antimicrobial resistance among PharmD students. Tropical Journal of Pharmaceutical Research. 2025;24(1):103-8.

122. Saksena R, Parida A, Jain M, Gaind R. Antibiotic use and antimicrobial resistance: Knowledge, Attitude and Practices survey of medical students to evaluate undergraduate training curriculum. Access Microbiol. 2025;7(1).

123. Swachia K, Jain C, Malhotra S, Bawa S, Beg MA, Bala S. Knowledge, Attitude and Practice regarding antimicrobial usage amongst interns and medical undergraduate students of a tertiary care teaching hospital. International Journal of Life Sciences Biotechnology and Pharma Research. 2025;14(2):715-22.

124. Akbulut ZencİRcİ S, KaragÖZ A, AkdenİZ KudubeŞ A, Baykan M. Evaluation of antimicrobial resistance awareness and related factors among university students in health-related departments. ESTUDAM Public Health Journal. 2025;10(2):163-71.

125. Sahib A, Abbas S, Hasson K, Mahmoud M. Experience of Antibiotic Use and Resistance Among Pharmacy Students in the University of Kerbala. Journal of Basic and Applied Research in Biomedicine. 2025;5(1):21-30.

126. Al-rudaini M, Anwar S, Al Sawaii H, Al Balushi R, Al Bahrani T, Al Kindi T, et al. Knowledge, Attitude, and Practice of Antibiotic Use among Medical Students in the College of Medicine, National University, Oman. Journal of Liaquat University of Medical & Health Sciences. 2025;24(02):195-9.

127. Orok E, Ikpe F, Williams T, Ekada I. Impact of educational intervention on knowledge of antimicrobial resistance and antibiotic use patterns among healthcare students: a pre- and post-intervention study. BMC Med Educ. 2025;25(1):283.

128. Babuya J, Waruingi D, Mungujakisa D, Ahimbisibwe O, Kako VR, Aporu F, et al. Medical students' knowledge, attitudes, and motivation towards antimicrobial resistance efforts in Eastern Uganda. PLoS One. 2025;20(2):e0314250.

129. Ijaz T, Noreen F, Iqbal F, Zeeshan M, Hammad M, Swati AAK. Lack of Antibiotic Knowledge and Misuse of Antibiotics by Medical Students in Lahore: A Cross-Sectional Study. Journal of Health, Wellness, and Community Research. 2025:e301-e.

130. Narasimha Murthy K, JN AH, Kumar N. Knowledge, attitudes, and practices toward antibiotic use and resistance among medical students: a cross-sectional study. International Journal of Basic & Clinical Pharmacology. 2025;14(5):760.

131. Rehman S, Sarfraz MR, Tariq H, Ismail A, Mansoor M, Salman A, et al. Antibiotic self-medication among healthcare students in Pakistan: A dual-center cross-sectional study. Work: Journal of Prevention, Assessment & Rehabilitation. 2025:10519815251382367.
